# Shared genetic architecture between autoimmune disorders and B-cell acute lymphoblastic leukemia: insights from large-scale genome-wide cross-trait analysis

**DOI:** 10.1186/s12916-024-03385-0

**Published:** 2024-04-15

**Authors:** Xinghao Yu, Yiyin Chen, Jia Chen, Yi Fan, Huimin Lu, Depei Wu, Yang Xu

**Affiliations:** 1 National Clinical Research Center for Hematologic Diseases, Jiangsu Institute of Hematology, The First Affiliated Hospital of Soochow University, Suzhou, China; 2 Collaborative Innovation Center of Hematology, Institute of Blood and Marrow Transplantation, Soochow University, Suzhou, China; 3 Department of Outpatient and Emergency, The First Affiliated Hospital of Soochow University, Suzhou, China

**Keywords:** Genetic overlap, B-cell acute lymphoblastic leukemia, Autoimmune disease, Mendelian randomization

## Abstract

**Background:**

To study the shared genetic structure between autoimmune diseases and B-cell acute lymphoblastic leukemia (B-ALL) and identify the shared risk loci and genes and genetic mechanisms involved.

**Methods:**

Based on large-scale genome-wide association study (GWAS) summary-level data sets, we observed genetic overlaps between autoimmune diseases and B-ALL, and cross-trait pleiotropic analysis was performed to detect shared pleiotropic loci and genes. A series of functional annotation and tissue-specific analysis were performed to determine the influence of pleiotropic genes. The heritability enrichment analysis was used to detect crucial immune cells and tissues. Finally, bidirectional Mendelian randomization (MR) methods were utilized to investigate the casual associations.

**Results:**

Our research highlighted shared genetic mechanisms between seven autoimmune disorders and B-ALL. A total of 73 pleiotropic loci were identified at the genome-wide significance level (*P* < 5 × 10^–8^), 16 of which had strong evidence of colocalization. We demonstrated that several loci have been previously reported (e.g., 17q21) and discovered some novel loci (e.g., 10p12, 5p13). Further gene-level identified 194 unique pleiotropic genes, for example *IKZF1*, *GATA3*, *IKZF3*, *GSDMB*, and *ORMDL3*. Pathway analysis determined the key role of cellular response to cytokine stimulus, B cell activation, and JAK-STAT signaling pathways. SNP-level and gene-level tissue enrichment suggested that crucial role pleiotropic mechanisms involved in the spleen, whole blood, and EBV-transformed lymphocytes. Also, hyprcoloc and stratified LD score regression analyses revealed that B cells at different developmental stages may be involved in mechanisms shared between two different diseases. Finally, two-sample MR analysis determined causal effects of asthma and rheumatoid arthritis on B-ALL.

**Conclusions:**

Our research proved shared genetic architecture between autoimmune disorders and B-ALL and shed light on the potential mechanism that might involve in.

**Supplementary Information:**

The online version contains supplementary material available at 10.1186/s12916-024-03385-0.

## Background

B-cell acute lymphoblastic leukemia (B-ALL) is a prevalent subtype of leukemia characterized by its highly malignant nature, primarily originating from the clonal expansion and abnormal proliferation of B lymphocytes within the hematopoietic system [1]. Autoimmune disorders are characterized by a disruption in self-tolerance, resulting in pathological alterations and clinical symptoms arising from immune responses targeting self-components [2]. Concurrently, the pathogenesis of several autoimmune disorders is intricately interwoven with the malfunctioning of B cells within the humoral immune system. The excessive activation of self-reactive B cells precipitates an overproduction of autoantibodies and immune complexes, which, in turn inflict damage upon a multitude of tissues and organs, culminating in the emergence of various autoimmune disorders [3]. To summarize, B cells assume a pivotal role in the orchestration of humoral immune responses, and their deregulation markedly contributes to the onset of autoimmune diseases and B-cell malignancies [4].

Epidemiological investigations have discovered associations between autoimmune disorders and B-cell malignancies. For example, rheumatoid arthritis (RA) patients exhibit a twofold increased risk of concomitant B-cell lymphomas when compared to their healthy counterparts [5]. In the case of systemic lupus erythematosus (SLE) and Sjögren’s syndrome patients, the risk amplifies significantly to 2.7–7.5 times [6] and 9–18 times [6], respectively. Previous studies observed that the standardized incidence ratio of ALL was estimated to be 2.77 after RA onset [7]. Studies also showed that at the time of diagnosis of malignancy, 15–30% of patients present with many of the typical features of rheumatic diseases [8]. However, current research focused primarily on the onset of autoimmune diseases on hematological malignancies risk, particularly diffuse large B-cell lymphoma and follicular lymphoma. This leaves a clear gap in understanding the pleiotropic mechanisms and bidirectional causations between B-ALL (a disease also derived from B lymphocytes) and autoimmune diseases. Only Li et al. have reported the shared mechanism between autoimmunity and B-ALL, specifically demonstrating the essential role of DYRK1a in mediating the noncanonical NF-κB activation induced by BAFF [9]. This underscores the existence of substantial knowledge gaps in this field, highlighting the urgent need to ascertain shared risk loci between these two disorders. It is worth noting that traditional clinical or epidemiological research may encounter challenges in ensuring the statistical effectiveness of such investigations.

**Fig. 1:**
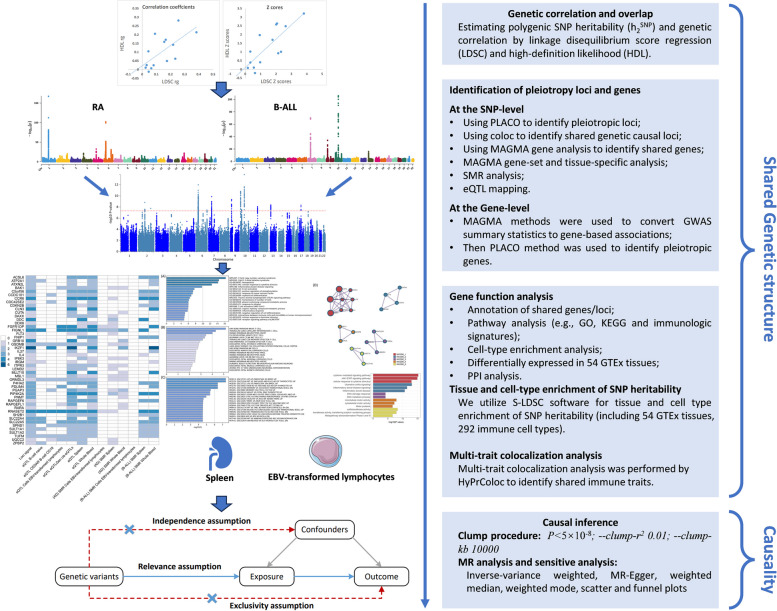
Study workflow

Recently, the linkage disequilibrium (LD) score regression (LDSC) approach has been developed to indicate whether there exists a genetic correlation between the two types of disease [10]. It is unclear whether the overall genetic correlation is attributable to a few loci or the entire genome. Few studies to date have systematically evaluated genetic overlap, shared susceptibility genes, and causality between autoimmune diseases and B-ALL. Cross-trait analyses that utilize the correlation of GWAS signals to study polyvalent genetic variants or loci between multiple traits have been shown to accurately identify shared loci between diseases or traits [11–13]. These pleiotropic loci can be targeted for intervention to potentially prevent or treat these diseases simultaneously. Recently, a novel method called “PLACO” was developed to identify pleiotropy at the SNP-level based on a level-α intersection–union test (IUT) [14]. Therefore, it is important to determine specific genetic variants or loci that lead to genome-wide genetic correlations or to delve into the shared genetic etiology of these two types of diseases. Our research flowchart is shown in Fig. 1.

## Methods

### GWAS summary data source

GWAS summary statistics for 16 autoimmune diseases were all publicly available from large-scale GWAS or GWAS meta-analyses: adult-onset asthma (AOA) [15, 16], childhood-onset asthma (COA) [15, 16], Graves’ disease (GD) [17, 18], Hashimoto’s disease (HD) [17, 18], hypothyroidism (HT) [17, 18], primary biliary cirrhosis (PBC) [19, 20], primary sclerosing cholangitis (PSC) [21, 22], inflammatory bowel disease (IBD) [23, 24], Crohn’s disease (CD) [23, 24], ulcerative colitis (UC) [23, 24], RA [25, 26], SLE [27, 28], multiple sclerosis (MS) [29, 30], systemic sclerosis (SS) [31, 32], type 1 diabetes (T1D) [17, 18], and vitiligo [33, 34]. GWAS summary statistics for B-ALL were generated in a meta-analysis of four GWAS including a total of 5321 cases and 16,666 controls of European ancestry [35, 36]. The same quality control procedure was followed for each study, the association between ALL status and SNP genotypes in each study was assessed using logistic regression, and genetic principal components were used as covariates in the association analysis. Risk estimates were finally combined by fixed-effects inverse variance weighted (IVW) meta-analysis. The sources and details of these datasets are summarized in Additional file 2: Table S1.

### Genetic overlap at the genome-wide level

We used LDSC to evaluate the genetic structure shared between autoimmune disorders and B-ALL [10]. The LD scores used in LDSC were calculated based on genotypes of common SNPs from European ancestry samples in the 1000 genomes project [37]. Standard errors (SE) were estimated by the jackknife method in LDSC which was further used to correct for attenuation bias. Intercept of LDSC was used to evaluate potential population overlaps between studies from different consortiums [10]. It is worth noting that no actual population overlap between autoimmune disorders and B-ALL studies existed in our analysis. A likelihood-based method, called high-definition likelihood (HDL), can utilize GWAS summary statistics to estimate genetic associations, which could reduce the variance of genetic association estimates by about 60% compared with the LDSC method [38].

We further investigated whether SNP heritability of autoimmune diseases and B-ALL was enriched in specific cells and tissues using hierarchical LDSC regression. Stratified-LDSC (S-LDSC) was applied to different immune cell data to assess whether specific cell types had significant genetic enrichment in these tissues. We downloaded 54 human tissues datasets from GTEx [39] and 292 immune cell types from the ImmGen consortium [40] (including B cells, γ δ T cells, α β T cells, innate lymphocytes, myeloid cells, stromal cells, and stem cells). After adjusting for the baseline model and all gene sets, we assessed the significance of the SNP heritability enrichment estimated in each tissue and cell by using the regression coefficient Z-scores and corresponding *P* values.

### Identification of pleiotropic loci and genes by using PLACO

A pleiotropic analysis under composite null hypothesis (PLACO) was used to identify pleiotropy among multiple autoimmune diseases and B-ALL at the SNP-level. SNPs reach genome-wide significant level (*P* < 5 × 10^–8^) and were viewed as pleiotropic variants. The functional mapping and annotation (FUMA) of GWAS was used to determine the genomic regions of these risk variants (i.e., pleiotropic loci) [41]. Also, a Bayesian colocalization analysis was conducted to determine the pleiotropic loci shared by autoimmune diseases and B-ALL [42]. To explore the shared mechanisms of the identified loci, nearby genes were mapped based on lead SNPs within each locus. Also, a generalized gene-set analysis of GWAS data (multi-marker analysis of genomic annotation, MAGMA) approach was used to determine the biological function of these pleiotropic loci. Specifically, we performed MAGMA gene analysis to identify pleiotropic genes by properly incorporating LD between markers and to detect multi-marker effects (*P* < 0.05/18,345 = 2.73 × 10^–6^) [43]. MAGMA gene-set analysis was performed to investigate the biofunction of lead SNPs [43], and a total of 10,678 gene sets including curated gene sets (c2.all) and go terms (c5.bp, c5.cc, and c5.mf) from Molecular Signatures Database (MSigDB) were finally tested [44]. Bonferroni correction was performed for all tested gene sets to avoid false positives (*P* < 0.05/10,678 = 4.68 × 10^–6^). Metascape webtools (metascape.org) performed a pathway enrichment analysis to determine the function of mapped genes based on MSigDB [44]. Genome-wide tissue-specific enrichment analysis was conducted based on 54 GTEx tissues [45] for the genome-wide pleiotropic results generated by PLACO. We also calculated the average expression (log_2_ transformed) of all identified pleiotropic genes in all 54 GTEx tissues [45] and tested tissue specificity by differentially expressed genes (DEGs) in each tissue (up- and down-regulated DEGs were precomputed by the signs of the t-statistics).

### Summary-based Mendelian randomization

Summary-based Mendelian randomization (SMR) [46] method combined summary-level data from GWAS with data from expression quantitative trait loci (eQTL) studies to identify genes whose expression levels are associated with complex traits due to pleiotropy. It employs SMR and HEIDI methods to test pleiotropic associations between gene expression levels and complex traits of interest using summary-level data from GWAS and eQTL studies. This approach could be interpreted as an analysis to test whether the magnitude of SNP effects on phenotype is mediated by gene expression.

### Multi-trait colocalization analysis

We utilized hypothesis prioritization for multi-trait colocalization (HyPrColoc) [47] method to perform multi-trait colocalization analysis to pinpoint the crucial roles that immune traits played in the onset of autoimmune disorders and B-ALL. Immune-wide GWAS data contains a total of 731 immune cells [48], which could be publicly available from the GWAS catalog (GCST0001391 ~ GCST0002121). Detailed information on the GWAS summary datasets for immune cells was added to Additional file 1: Supplementary Methods.

### Causal association analysis

We performed a one-directional two-sample Mendelian randomization (MR) analysis to assess possible causal effects of autoimmune disorders on B-ALL risk. The “clumping” procedure in PLINK 1.9 software was used to extract independent significance SNPs for all autoimmune diseases (*P* < 5 × 10^–8^), where *r*^2^ was set to 0.001 and window size was set to a physical distance of 10,000 KB [49]. Notably, *r*^2^ was calculated based on the 1000 genomes project phase 3 as a reference panel. Proportion of variance explained (PVE) and *F* statistic (*F* > 10) was used to measure the strength of instrumental variables (IVs) (see Additional File 1: Supplementary Methods) [50]. To verify causality among these trait pairs, six MR approaches were performed with each set of IVs, i.e., IVW, Debiased-IVW (DIVW) [51], weighted median approach [52], MR pleiotropy residual sum and outlier (MR-PRESSO) [53], MR-Egger [54], MR robust adjusted profile score (MR-RAPS) [55], and mode-based estimate [56] method. Cochran’s *Q* statistics was used to examine the effect size heterogeneity across the IVs (see Additional File 1: Supplementary Methods) [57, 58]. Additionally, the intercept of MR-Egger regression and global test of MR-PRSSO were utilized to detect horizontal pleiotropy [53, 54]. Detailed information on used MR methods was described in Additional file 1: Supplementary Method.

### Software and packages

The main statistical analysis was performed in R (version 3.5.3). LDSC and S-LDSC analysis were implemented with “LDSC” software (v1.0.1) [10]. PLACO was performed with “PLACO” package [14]. Bayesian colocalization analysis was performed with the “coloc” package (version 5.2.1) [42] and HyPrColoc was performed with the “hyprcoloc” package (version 1.0) [47]. Function analysis was performed by FUMA web tool [41]. MAGMA gene and gene-set analysis were performed by MAGMA software [43]. Two-sample MR analysis was conducted with “MendelianRandomization” (version 0.9.0) [59], mr.raps (version 0.4.1) [55], and MRPRESSO (version 1.0) [53] packages. A copy of the main code used in this research is available at: https://github.com/biostatYu/MRcode-/tree/main/AD_BALL. 

## Results

### Shared genetic architecture between autoimmune disorders and B-ALL

We first evaluated the genetic correlation between autoimmune diseases and B-ALL and results from the LDSC and HDL methods were highly consistent (Table 1 and Additional file 2: Table S2). Specifically, by using the LDSC method, six traits were identified to be genetically correlated with B-ALL, including AOA, HT, IBD, CD, RA, and MS. While implementing the HDL method, significant genetic correlations were observed among AOA, HT, PBC, RA, MS, and B-ALL, leading to a final union set of seven pairwise traits for further analysis. However, we did not find significant genetic correlation between IBD and CD and HDL results. It was noting that only RA remained significantly genetical correlated with B-ALL risk after applying the Bonferroni correction (*P* = 0.003 < 0.05/16).

**Table 1:** Genetic correlation between autoimmune diseases and B-ALL

Trait pairs	LDSC		HDL	
	*r*_*g*_ (SE)	*P*	*r*_*g*_ (SE)	*P*
B-ALL&AOA	0.156 (0.078)	*0.045*	0.159 (0.062)	*0.010*
B-ALL&COA	0.046 (0.073)	0.529	− 0.012 (0.056)	0.835
B-ALL&GD	0.025 (0.151)	0.871	0.012 (0.133)	0.931
B-ALL&HD	0.256 (0.145)	0.077	0.281 (0.165)	0.089
B-ALL&HT	0.167 (0.081)	*0.039*	0.171 (0.065)	*0.008*
B-ALL&PBC	0.091 (0.097)	0.350	0.205 (0.104)	*0.049*
B-ALL&PSC	− 0.072 (0.128)	0.573	/	/
B-ALL&IBD	0.175 (0.082)	*0.033*	0.05 (0.053)	0.353
B-ALL&CD	0.234 (0.101)	*0.020*	0.063 (0.062)	0.311
B-ALL&UC	0.081 (0.084)	0.335	0.025 (0.062)	0.682
B-ALL&RA	0.383 (0.101)	*1* × *10*^*–4*^	0.214 (0.067)	*1.32* × *10*^*–3*^
B-ALL&MS	0.219 (0.084)	*0.009*	0.142 (0.057)	*0.013*
B-ALL&SS	0.212 (0.166)	0.203	/	/
B-ALL&SLE	0.064 (0.117)	0.581	0.008 (0.076)	0.917
B-ALL&T1D	0.044 (0.137)	0.746	0.105 (0.102)	0.305
B-ALL&Vitiligo	0.032 (0.097)	0.745	0.026 (0.066)	0.693

*LDSC* linkage disequilibrium score regression, *HDL* high-definition likelihood, *SE* standard error, *B-ALL* B-cell acute lymphoblastic leukemia, *AOA* adult-onset asthma, *COA* childhood-onset asthma, *GD* Grave’s disease, *HD* Hashimoto’s disease, *HT* hypothyroidism, *PBC* primary biliary cirrhosis, *PSC* primary sclerosing cholangitis, *IBD* inflammatory bowel disease, *CD* Crohn’s disease, *UC* ulcerative colitis, *RA* rheumatoid arthritis, *MS* multiple sclerosis, *SS* systemic sclerosis, *SLE* systemic lupus erythematosus, *T1D* type 1 diabetes

### Pleiotropic loci and genes identified for multiple autoimmune disorders and B-ALL

**Fig. 2:**
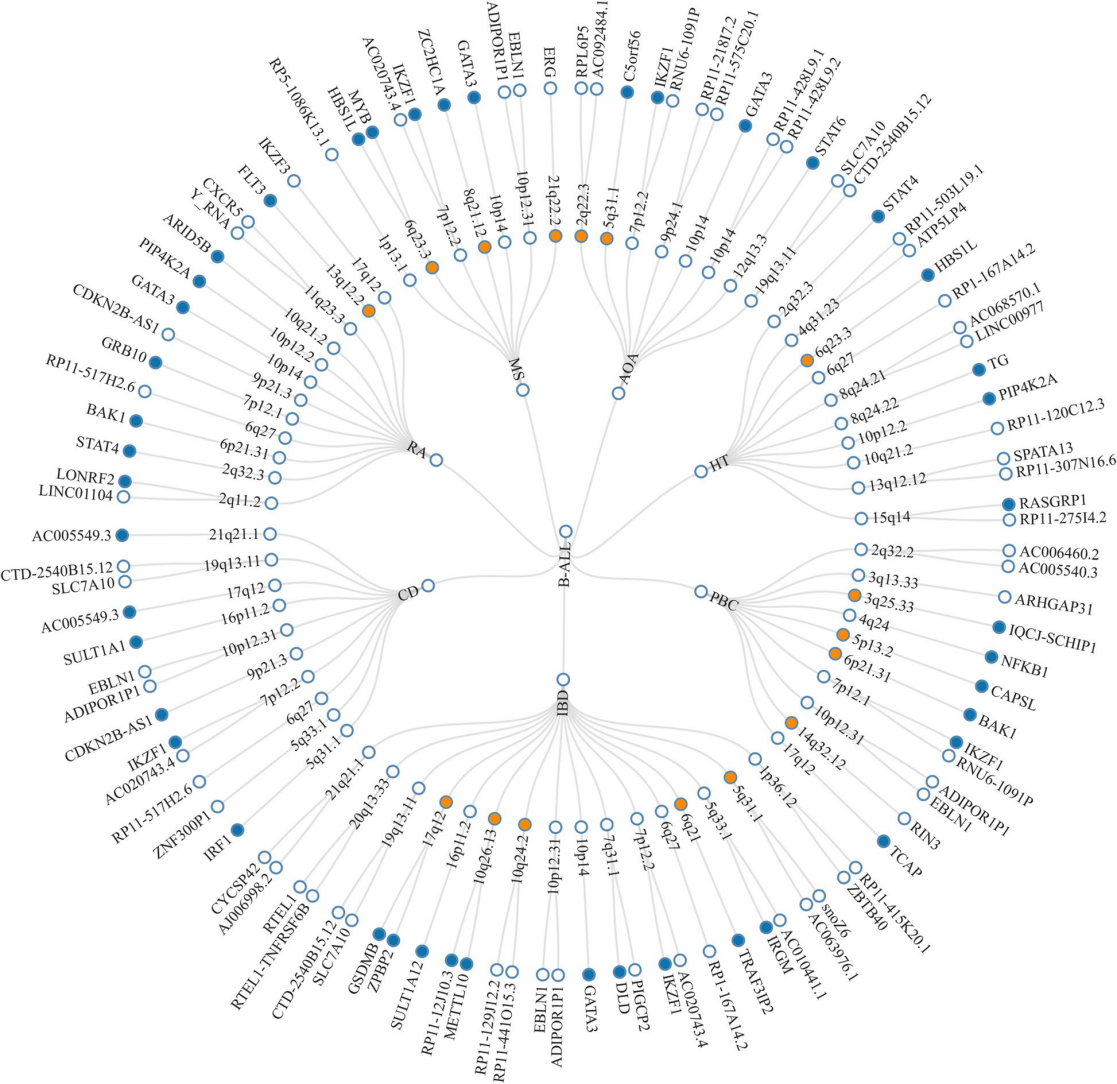
The circular diagram presents pleiotropic loci and genes identified by PLACO among seven trait pairs. Note: Shared loci identified by colocalization analysis are highlighted in orange; shared genes identified by MAGMA analysis are highlighted in blue. B-ALL B-cell acute lymphoblastic leukemia, AOA adult-onset asthma, HT hypothyroidism, PBC primary biliary cirrhosis, IBD inflammatory bowel disease, CD Crohn’s disease, RA rheumatoid arthritis, MS multiple sclerosis

**Fig. 3:**
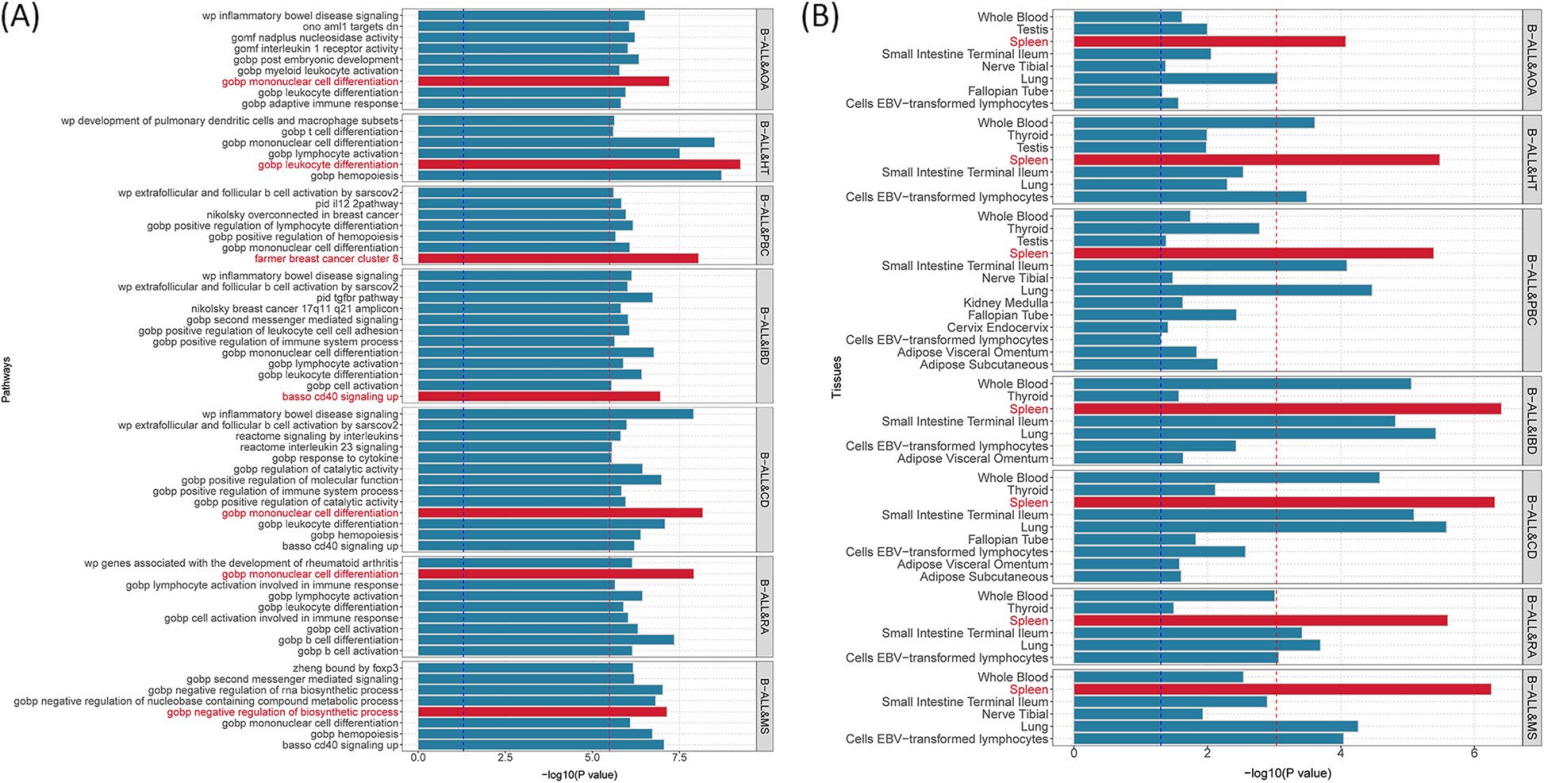
Bar plot of MAGMA gene-set (**A**) and tissue-specific (**B**) analysis for genome-wide pleiotropic results. Note: The red dotted line represents the significance of 0.05 after multiple corrections, and the blue represents the significance of 0.05. B-ALL B-cell acute lymphoblastic leukemia, AOA adult-onset asthma, HT hypothyroidism, PBC primary biliary cirrhosis, IBD inflammatory bowel disease, CD Crohn’s disease, RA rheumatoid arthritis, MS multiple sclerosis

Given the shared genetic mechanisms between autoimmune diseases and B-ALL identified by LDSC and HDL, we used novel pleiotropy analyses (PLACO) to identify potential pleiotropic loci for both diseases (Additional file 1: Fig. S1). The QQ plots demonstrated no premature divergence between observed and expected values, ruling out the possibility of group stratification (Additional file 1: Fig. S2). Based on PLACO results, we identified a total of 73 pleiotropic genomic risk loci associated with both autoimmune disorders and B-ALL using FUMA (*P* < 5 × 10^–8^) (Fig. 2, Additional file 1: Fig. S1, and Additional file 2: Table S3). Colocalization analysis finally identified 16 of 73 (21.9%) potential pleiotropic loci with PP.H4 greater than 0.7 (e.g., 5p13) (Table 2). The regional plots for each trait pair are presented in Additional file 1: Fig. S3 ~ S8. Notably, some pleiotropic regions were shared between different pairs, for example, genome regions 7p12.2, 10p14, 6q27, and 10p12.31 were identified in four pairs (Additional file 2: Table S4). The MAGMA analysis of gene sets suggested that the identified pleiotropic loci may participate in the control of the immune system, hematopoiesis, and various other processes (Fig. 3A and Additional file 2: Table S5). Notably, significant monocyte differentiation pathway was found for all trait pairs, and significant leukocyte differentiation was found for all five trait pairs. Further tissue-specific analysis found these risk loci were enriched in several immune-related tissues (e.g., spleen, whole blood, Epstein–Barr virus (EBV)-transformed lymphocytes) (Fig. 3B and Additional file 2: Table S6). ANNOVAR category annotation showed that 28 of 73 lead SNPs (38.4%) were intronic variants and 30 of 73 (41.1%) were intergenic variants. Only 2 of 73 (3%) lead SNPs were exonic variants (Additional file 2: Table S3).

**Table 2:** 16 colocalized loci identified by colocalization analysis performed on 73 pleiotropic loci (PP.H4 > 0.7)

Trait pairs	Locus boundary	Region	Nearest genes	Lead SNPs	*P*	PP H4
B-ALL&AOA	2:145800791–146642994	2q22.3	RPL6P5, AC092484.1	rs12992327	2.61 × 10^–08^	0.772
B-ALL&AOA	5:130812049–132489413	5q31.1	C5orf56	rs11741255	3.61 × 10^–13^	0.798
B-ALL&HT	6:135176498–135536586	6q23.3	HBS1L	rs2210366	1.84 × 10^–08^	0.854
B-ALL&IBD	5:130437485–132319450	5q31.1	snoZ6, AC063976.1	rs27437	2.66 × 10^–13^	0.713
B-ALL&IBD	6:111398304–112528738	6q21	TRAF3IP2	rs33980500	2.61 × 10^–09^	0.942
B-ALL&IBD	10:100601902–101268250	10q24.2	RP11-441O15.3, RP11-129J12.2	rs2490285	2.66 × 10^–08^	0.903
B-ALL&IBD	10:126192582–126590319	10q26.13	METTL10, RP11-12J10.3	rs11245358	2.36 × 10^–08^	0.907
B-ALLIBD	17:37486160–38384187	17q12	ZPBP2, GSDMB	rs117504211	1.47 × 10^–08^	0.961
B-ALL&MS	6:135195857–135581900	6q23.3	HBS1L, MYB	rs9494168	1.39 × 10^–08^	0.953
B-ALL&MS	8:78606328–79845682	8q21.12	ZC2HC1A	rs10102877	1.97 × 10^–09^	0.884
B-ALL&MS	21:39835031–39945134	21q22.2	ERG	rs2836438	5.16 × 10^–09^	0.958
B-ALL&PBC	3:159455579–159818194	3q25.33	IQCJ-SCHIP1	rs13072356	1.41 × 10^–08^	0.742
B-ALL&PBC	5:35078698–36482458	5p13.2	CAPSL	rs115727000	3.54 × 10^–08^	0.848
B-ALL&PBC	6:33187355–33806276	6p21.31	BAK1	rs210134	8.03 × 10^–10^	0.981
B-ALL&PBC	14:93049865–93131795	14q32.12	RIN3	rs72699846	8.64 × 10^–10^	0.734
B-ALL&RA	13:28490592–28665187	13q12.2	FLT3	rs9512977	4.3 × 10^–09^	0.872

Lead SNP was the SNP with minimum *P* values within the corresponding locus. PP.H4 was the posterior probability of H4 calculated by coloc analysis; the Locus boundary was defined as “chromosome: start–end”

*PP.H4* the posterior probability of H4, *B-ALL* B-cell acute lymphoblastic leukemia, *AOA* adult-onset asthma, *HT* hypothyroidism, *PBC* primary biliary cirrhosis, *IBD* inflammatory bowel disease, *CD* Crohn’s disease, *RA* rheumatoid arthritis, *MS* multiple sclerosis

### Pleiotropic genes identified and enrichment analysis

**Fig. 4:**
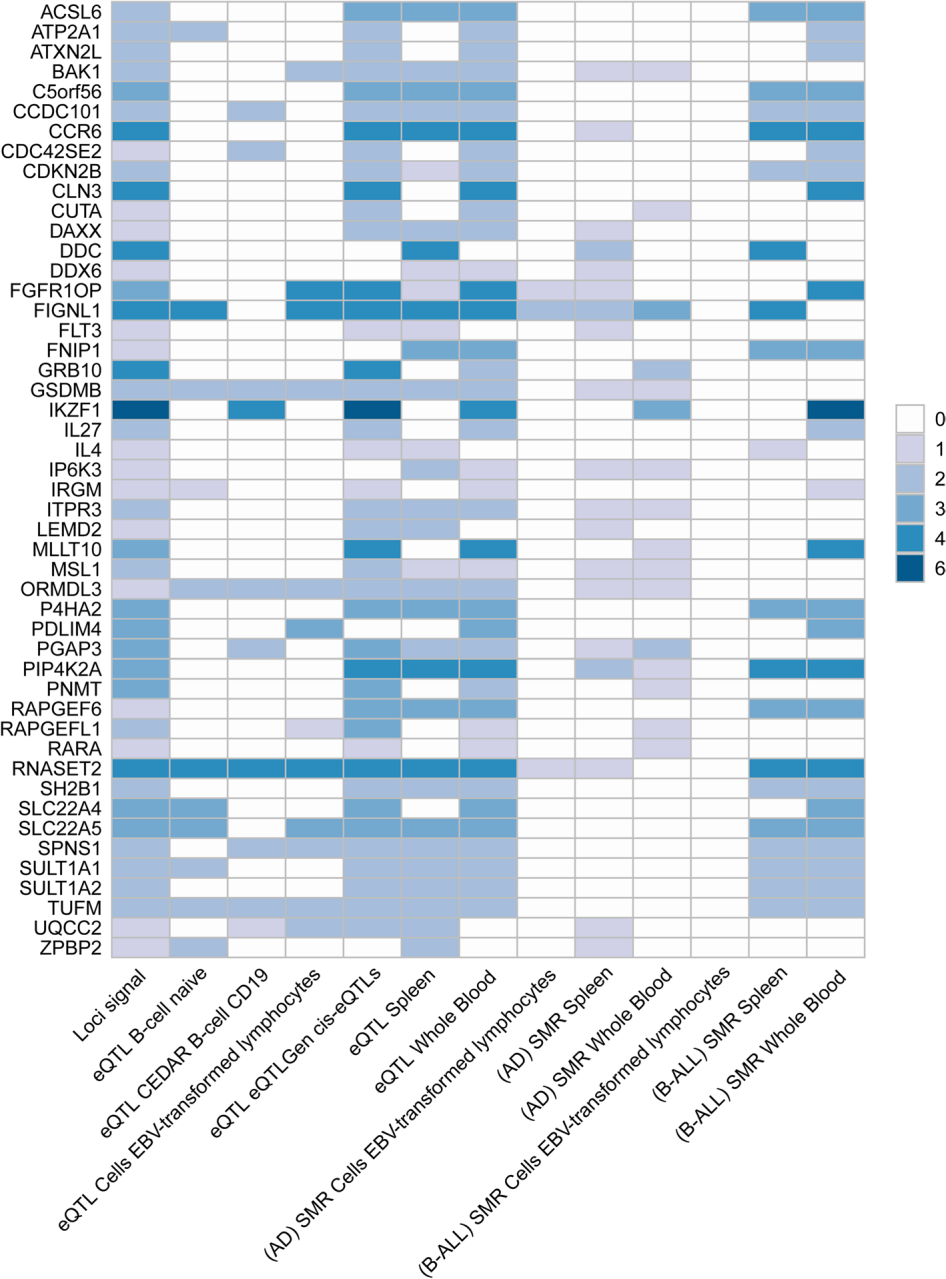
Overview of pleiotropic genes (highlighted in all three signals) for the autoimmune disorders and B-ALL. Note: The signals represent hits of genes across different trait pairs. eQTL expression quantitative trait loci, SMR summary-based Mendelian randomization, AD autoimmune disorders, B-ALL B-cell acute lymphoblastic leukemia, AOA adult-onset asthma, HT hypothyroidism, PBC primary biliary cirrhosis, IBD inflammatory bowel disease, CD Crohn’s disease, RA rheumatoid arthritis, MS multiple sclerosis

We used different methods to map the identified SNP-level signals into the gene-level signals. By using MAGMA gene analysis, a total of 341 significant pleiotropic genes were determined as pleiotropic genes between multiple autoimmune diseases and B-ALL (194 unique) (Additional file 2: Table S7 and Additional file 1: Fig. S9). Additional file 2: Table S8 lists the details of these genes. MAGMA gene analysis detected 92 repeated pleiotropic genes across different trait pairs, with *IKZF1* identified as a pleiotropic gene for six pairs, followed by *MLLT10*, *FIGNL1*, *RNASET2*, *CCR6*, *GATA3*, *CLN3*, *PIP4K2A*, *DDC*, *RP11-514O12.4*, *FGFR1OP*, and *GRB10* in four trait pairs. eQTL analysis identified multiple hits of *IKZF1* in blood- and immune-related tissues (e.g., naïve B cell, CD19 B-cell, EBV-transformed lymphocytes cells, cis-eQTLs, trans-eQTLs, spleen, whole blood). Five genes (i.e., *TUFM*, *ZC2HC1A*, *RNASET2*, *GSDMB,* and *ORMDL3*) were observed to be significant in five different tissues. We summarized the landscape of pleiotropic genes identified in different methods and tissues in Fig. 4. We observed several genes (*RNASET2* and *FIGNL1*) were significantly mapped in different tissues with different methods. The *IKZF1* gene was also highlighted in whole blood tissues (Fig. 4).

**Fig. 5:**
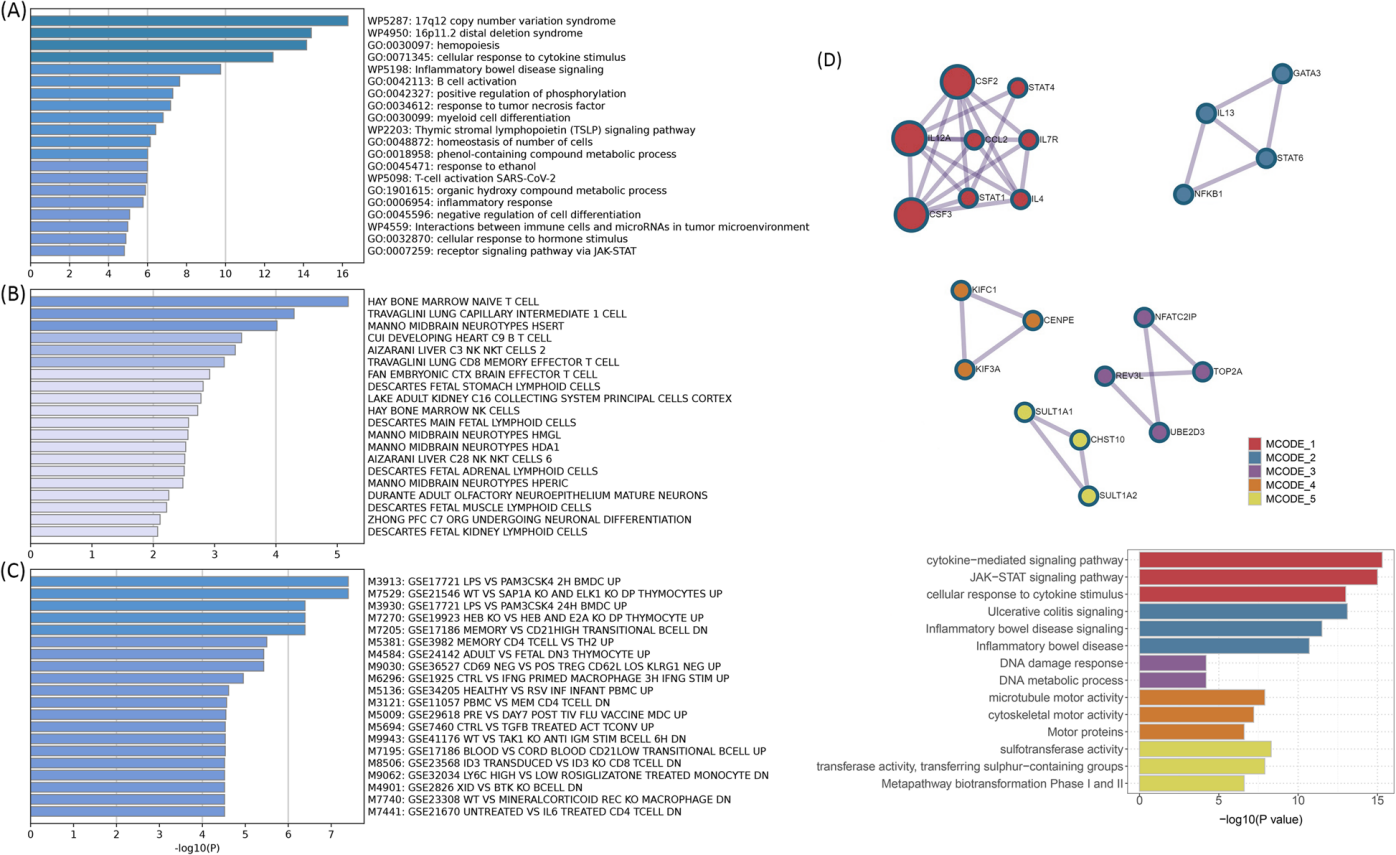
**A** Pathway enrichments for identified pleiotropic genes (KEGG, GO, Wiki pathways). **B** Cell-type enrichments for identified pleiotropic genes. **C** Immune signatures enrichments for identified pleiotropic genes. **D** Protein–protein interaction analysis based on identified pleiotropic genes

The shared mechanism between autoimmune diseases and B-ALL may involve specific organs or tissues involvement. Numerous genes (e.g., *TOP2A*, *IKZF3*, *MYB,* and *CD80*) showed significant differential expression in EBV-transformed lymphocytes, and *APOBR*, *IKZF1,* and *IL7R* showed significant differential expression in spleen and whole blood tissues (Additional file 1: Fig. S10 and Additional file 2: Table S9). Tissue enrichment analysis showed that these genes were also enriched into the spleen and EBV-transformed lymphocytes (Additional file 1: Fig. S11 and Additional file 2: Table S10). Additional S-LDSC based on multiple tissues identified significant SNP heritability enrichment for all autoimmune diseases (except AOA) in each of the monocytes, blood cells, and spleen, after adjusting for the baseline model (Additional file 1: Fig. S12 and Additional file 2: Table S11). Further enrichment analysis of the GO biological processes associated with these genes indicated higher enrichment in the cellular response to cytokine stimulation, B cell activation, response to tumor necrosis factor, inflammatory response, and receptor signaling pathway via JAK-STAT (Fig. 5A). These pathways play important roles in immune regulation and leukemogenesis. Cell type enrichment analysis showed the highest significance for bone marrow naïve T cells (Fig. 5B). Furthermore, we found that these genes were numerically enriched in several immunologic signatures (e.g., MEMORY VS CD21HIGH TRANSITIONAL BCELL DN) (Fig. 5C). The PPI analysis showed that five PPI networks were constructed, including the JAK-STAT signaling pathway and multiple pathways related to DNA damage were involved. And 22 proteins (e.g., STAT, NFKB1, and GATA3) could participate in these pathways (Fig. 5D). Also, the results suggest that heritability is enriched in the blood, EBV-transformed lymphocytes, whole blood, and palatine tonsil tissues among five or more autoimmune diseases and B-ALL.

### Immune-related mechanisms shared between autoimmune disorders and B-ALL

The shared mechanism involving affected tissues such as the spleen, lymphocytes, and whole blood, suggested an important involvement of immune mechanisms in the inter-disease. We used the S-LDSC method to determine the heritability enrichment of pleiotropy in immune cells and the HyPrColoc method to identify immune cells with co-localization signals with pleiotropic motifs. S-LDSC observed heritability enrichment of B cells in both autoimmune diseases and B-ALL. When analyzing the enrichment of immune traits from ImmGen, we also observed that two cell traits in the B cell panel were enriched: B.FrE.BM (CD19^+^IgM^+^AA4.1^+^HSA^+^) and preB.FrD.BM (CD19^+^IgM^−^CD45R^+^CD43^−^). Additionally, numerous cell traits in the T cell panel were also identified, implying the potential immune mechanisms shared (Additional file 1: Fig. S12 and Additional file 2: Table S11). Then multi-trait colocalization analysis by using HyPrColoc was performed to pinpoint key immune cells (Additional file 2: Table S12). Results highlight 59 pleiotropic loci, of which 19 were unique, and these loci support the important role of 42 unique immune cells in autoimmune diseases and B-ALL by sharing causal variants. Our results support the critical influence of BAFF-R, CD4, CD45, and CD28 on different cells. Notably, a total of six BAFF-R-related immune traits were observed, including BAFF-R on B cell, BAFF-R on CD20^−^, BAFF-R on CD24^+^ CD27^+^, BAFF-R on IgD^+^ CD24^−^, BAFF-R on IgD^+^ CD24^+^, and BAFF-R on IgD^+^ CD38^−^. Interestingly, BAFF-R on B cell and BAFF-R on CD24^+^ CD27^+^ were both shared among three trait pairs (i.e., B-ALL&IBD, B-ALL&PBC, B-ALL&RA).

### The causal relationship between autoimmune diseases and B-ALL estimated by MR

**Fig. 6:**
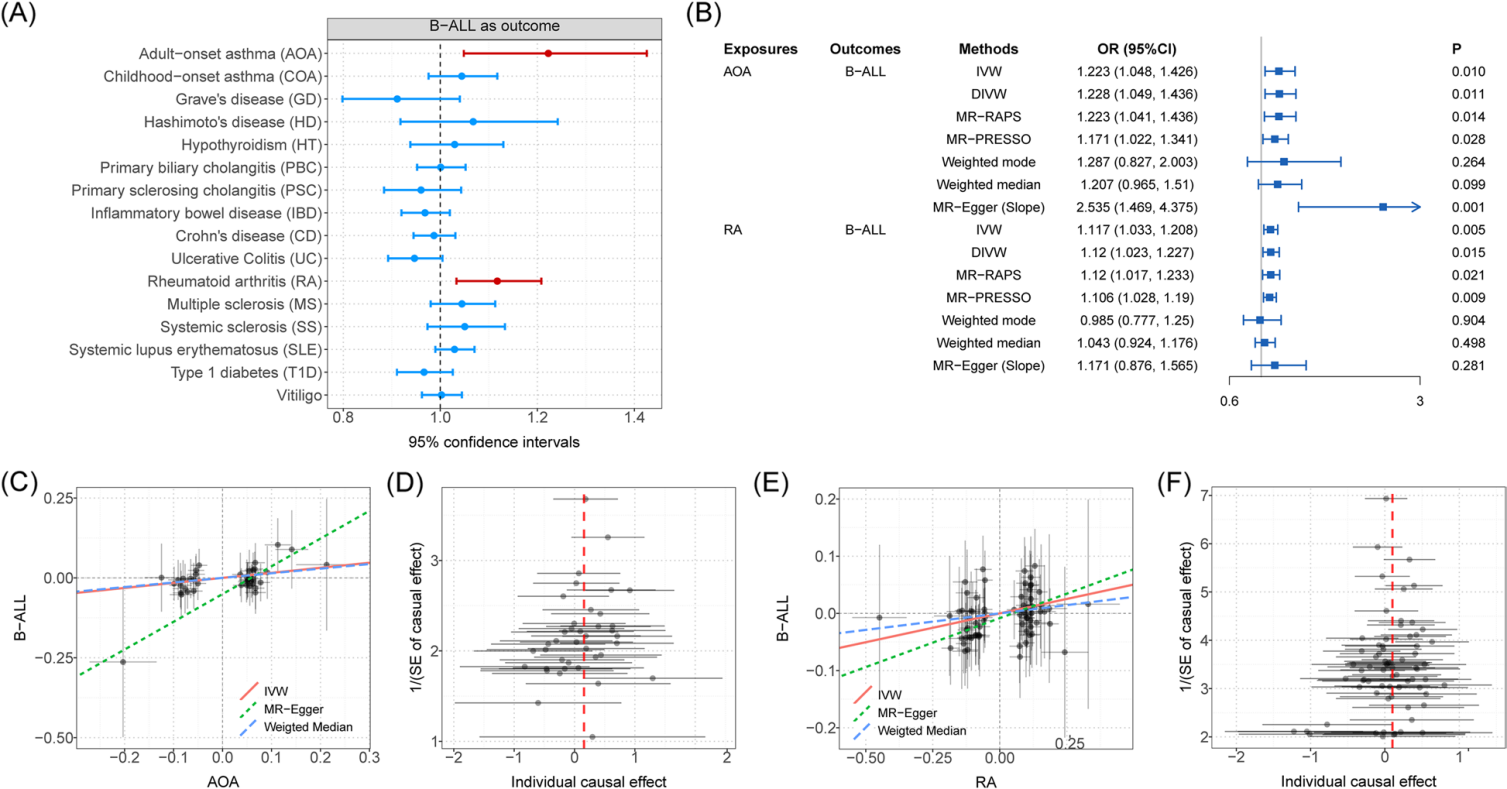
**A** The forest plot shows causal associations between autoimmune disorders and B-ALL by using one-directional MR analysis. Note: Causal effects were estimated by using IVW method. **B** Forest plot shows causal effects of AOA and RA on B-ALL risk estimated by using different methods. **C** Scatter plot shows significant causal association between AOA and B-ALL risk. **D** Funnel plot shows significant causal association between AOA and B-ALL risk. **E** Scatter plot shows significant causal association between RA and B-ALL risk. **F** Funnel plot shows significant causal association between RA and B-ALL risk. Associations highlighted with red represent that associations were significant in more than three main MR methods

MR analyses using the IVW method showed significant positive associations between two autoimmune diseases (AOA and RA) and B-ALL risk (Fig. 6A and Additional file 2: Table S13). The risk of B-ALL was found to be able to be increased as the risk of AOA increases, the effect size was estimated by using the IVW method (OR = 1.223, 95%CI = 1.048 ~ 1.426, *P* = 0.010). Another four methods (DIVW, MR-RAPS, MR-PRESSO, and slope of MR-Egger) are consistent with the results of the IVW method. Although a significant intercept of MR-Egger might indicate the existence of potential horizontal pleiotropy, the global test of MR-PRESSO ruled out this possibility (*P* = 0.632). We also observed significant causal effects of RA onset on B-ALL risk by using the IVW method (OR = 1.117, 95%CI = 1.033 ~ 1.208, *P* = 0.005). DIVW, MR-RAPS, and MR-PRESSO support this association (Fig. 6B), where the intercept of MR-Egger and the global test of MR-PRESSO ruled out the possibility of horizontal pleiotropy (Additional file 2: Table S14). Additional scatter and funnel plots eliminate the possibility of potential outliers (Fig. 6C–D). However, after the Bonferroni adjustment, no causal associations between autoimmune disorders and B-ALL remained statistically significant (*P* = 0.003 < 0.05/16). Finally, reverse MR analysis ruled out the possibility of reverse-directional causality.

## Discussion

Given the critical contribution of B cells to autoimmune disorders and B-ALL, there may be a complex relationship between them [60]. The study employed comprehensive genetic methodologies to investigate the genetic correlation between autoimmune disorders and B-ALL. The study determined pleiotropic loci using cross-trait PLACO analysis and identified pleiotropic genes through the MAGMA method. Then the key pathways and immunological mechanisms involved were identified. Finally, comprehensive MR analysis and sensitive analysis established the causal relationships between autoimmune diseases and B-ALL.

Through genetic correlation analysis, we observed significant genetic overlap between B-ALL and seven autoimmune disorders, including AOA, HT, IBD, CD, PBC, RA, and MS. We provide strong evidence for a shared genetic mechanism between RA and B-ALL, as well as MR evidence suggesting that patients with RA symptoms should be alerted to the risk of progression to ALL, which is consistent with previous studies [7, 8]. Additionally, study have shown that 34 of 699 ALL patients diagnosed and followed had previously received varying doses of steroids for aplastic events or arthritis-based rheumatic diseases [61]. By using genetic variables, MR methods could well avoid the influence of possible confounding factors. Therefore, we believe that in addition to the effect of immunosuppressants, RA itself will also play an important role in the risk of B-ALL. We also observed significantly causal effects of AOA on B-ALL risk, which was ambiguous in previous studies: a systematic review supported the protective effect of asthma on ALL [62], two types of research included showed significant high risks of ALL in patients with a history of asthma [63, 64].

We identified a series of genetically risk loci associated with both autoimmune diseases and B-ALL, and some of which were observed in multiple phenotype pairs (e.g., 7p12.2, 10p14, 6q27, 10p12.31). Previous studies gave the evidence of key role these loci played in the development of autoimmune disorders and B-ALL. For example, loci on 7p12.2 (*IKZF1*) had been proven to be associated with risk of childhood B-ALL [65], which was also identified as susceptibility genes for SLE [66]. After searching for the GWAS catalog, 7p12.2 had been reported to be associated with multiple autoimmune disorders, including CD [23], IBD [23], RA [25], and MS [67]. *GATA3* (10p14) is a key regulator in the immune system, especially in the differentiation and function of type 2 helper (Th2) cells [68]. Th2 cells have been demonstrated to play a role in various autoimmune diseases, including SLE and IBD [69, 70]. Recent research also highlighted the role of noncoding genetic variation (rs3824462) in *GATA3*, linking it to an increased risk of Ph-like ALL, a common subtype of B-ALL. The study revealed that rs3824462 induced local and global changes in chromatin conformation, activating JAK-STAT pathway and promoting disease development [71].

We searched for the identified risk loci in the GWAS catalog (last update in 2023 December 20) [72] and found that some of the risk loci have been reported to be associated with both B-ALL and autoimmune disorders (Additional file 1: Fig. S13 and Additional file 2: Table S15). For instance, the 17q21 locus is implicated in various autoimmune diseases, including asthma [73, 74], IBD [75, 76], T1D [77], and SLE [78]. This locus, housing *IKZF3*, *GSDMB*, and *ORMDL3*, involved in lymphocyte development [79], pyroptosis [80], and inflammatory response [81], has been challenging to dissect. *GSDMB* and *ORMDL3* represent the target genes of rs2290400, and its minor allele is associated with a protective effect against ALL [82]. *IKZF3* polymorphism contributes to B-ALL with a 1.5-fold to twofold increase in relative risk [83]. Genes previously reported to be associated with leukemia have also been observed in our results to be correlated with autoimmune diseases: *MLLT10* (10p12) participates in various chromosomal rearrangements associated with ALL and acute myeloid leukemia (AML) [84]. It is implicated in chromatin structure regulation and DNA damage response, deemed crucial for early development, maintenance, and differentiation of hematopoietic stem cells. While direct evidence for the impact of *MLLT10* on autoimmune diseases has not been established, studies indicated a close association with C-reactive protein levels [85], widely recognized as a valuable indicator of disease activity in various autoimmune rheumatic diseases [86]. Simultaneously, certain genes previously reported to be associated with autoimmune disorders have also been found in our results to be associated with B-ALL. *IRGM* (5q33) encodes a member of the immunity-related GTPase family, crucial in innate immunity and inflammatory responses [87]. Previous studies have linked *IRGM* to CD [88–90], UC [91], and IBD [23]. *CAPSL* (5p13) has been reported to be associated with PBC [92], T1D [93], asthma [94], and SLE [95]. Although direct evidence of its association with ALL is lacking, increased mRNA levels have been observed in AML patients [96]. Additionally, the long non-coding RNA *C5orf56* (5q31) has been identified for its protective role in IBD [97]. *SCHIP1* (3q25) has been associated with SLE [98], while *RNASET2*(6q27) has been identified as a risk gene for both vitiligo [99] and GD [100].

Shared genetic structures observed in our research revealed common mechanisms between autoimmune disease and B-ALL. Identified genes were observed to participate in several pathways, like B cell activation, cellular response to cytokine stimulus, and inflammatory response. For each disease pair, we observed a significant enrichment of pleiotropy to the spleen, a critical site for B cell development. Notably, a substantial presence of BAFF-R-associated immune signature, a key regulator of B cell function and survival, was discerned in a multi-trait colocalization analysis. These findings collectively underscored the pivotal role played by B cells in both autoimmune disorders and B-ALL. In autoimmune conditions, B cells are exposed to antigens, undergo activation, and subsequently proliferate and expand clonally, thereby increasing the risk of accumulating genetic mutations, and finally leads to the emergence and progression of B-ALL [60]. We can think that *ORMDL3* and *IKZF3*, mentioned earlier, play crucial roles in this context, as evidenced by prior literature reporting ORMDL3’s vital role in B cell survival [101], and *IKZF3*’s predominant regulation of B cell differentiation, activation response, and proliferation [102]. Furthermore, malignancies arising from B cells consistently exhibit concurrent autoimmune disorders at any stage, whereas those derived from T cells are less commonly linked to autoimmune phenomena [103]. Nevertheless, our findings also identified numerous cell traits in the T cell panel, and we speculate that this may be attributed to interactions between B and T cells. The JAK-STAT pathway may represent a crucial mechanism in this context, as it has been targeted in autoimmune diseases [104] and its role in B-ALL involves the disruption of preleukemic cells differentiation [105]. Our results highlighted the critical role of EBV infection as a trigger for both autoimmune disorders and B-ALL: tissue-specific analysis revealed enriched risk loci in EBV-transformed lymphocytes, and the central role of *IKZF1* in this cell was also identified by gene-level analyses. EBV remains latent in memory B cells after infection, and reactivation can induce B cell clonal immortalization, promoting lymphomagenesis [106]. Additionally, EBV-induced autoimmunity has been reported to increase the risk of autoimmune diseases [107].

### Limitations

Our study is not without limitations. Firstly, as with other similar studies, the data used in this study was summary-level, and individual-level datasets were not available. Further stratification of the population (e.g., gender, age, etc.) was therefore not possible. Secondly, the sample size of immune cell GWAS used in this study was limited. Therefore, caution should be exercised in interpreting the role of immune cells and drawing conclusions in our studies. Thirdly, it should be noted that our study was limited to European ancestry and may not be generalizable to other ancestries. It is important to be equally cautious in concluding our findings since the relatively small sample size of B-ALL may result in limited statistical power.

## Conclusions

Our research has uncovered the intricate connections between autoimmune disorders, especially AOA, HT, IBD, CD, RA, and MS and B-ALL. Identification of pleiotropic risk loci (7p12, 10p14, 6q27, and 10p12) and genes (*IKZF1*, *GATA3*, *IKZF3*, *GSDMB*, and *ORMDL3*) shared between diseases suggested shared mechanisms, such as B cell activation and JAK-STAT pathway, common triggers like EBV infection. Additionally, our findings have shed light on and the causal links between autoimmune disorders (AOA and RA) and B-ALL.

### Supplementary Information

**Additional file 1:** Supplementary Methods and **Fig. S1-S3.** Supplementary Methods - A supplementary document on GWAS quality control, PLACO method, colocalization analysis, MAGMA analysis, HyPrColoc method, immune cell data description, and Mendelian randomization analysis. **Fig. S1.** Manhattan plot of the PLACO results. **Fig. S2.** -QQ plots for pleiotropic results performed by PLACO. **Fig. S3.** Regional plots of each colocalized locus (PP.H4 > 0.7) identified for corresponding trait pair (B-ALL&AOA) by using the PLACO. **Fig. S4.** Regional plots of each colocalized locus (PP.H4 > 0.7) identified for corresponding trait pair (B- B-ALL&HT) by using the PLACO. **Fig. S5.** Regional plots of each colocalized locus (PP.H4 > 0.7) identified for corresponding trait pair (B-ALL&PBC) by using the PLACO. **Fig. S6.** Regional plots of each colocalized locus (PP.H4 > 0.7) identified for corresponding trait pair (B-ALL&IBD) by using the PLACO. **Fig. S7.** Regional plots of each colocalized locus (PP.H4 > 0.7) identified for corresponding trait pair (B-ALL&MS) by using the PLACO. **Fig. S8.** Regional plot of each colocalized locus (PP.H4 > 0.7) identified for corresponding trait pair (B- B-ALL&RA) by using the PLACO. **Fig. S9.** Manhattan plot of MAGMA gene analysis. **Fig. S10.** Heatmap for expression values of pleiotropic genes in different tissues identified by MAGMA analysis. **Fig. S11.** Gene-set enrichment for identified pleiotropic genes. Red panels represent significant tissues after Bonferroni adjustment. **Fig. S12.** Heatmap of tissues and immune traits shared between autoimmune disorders and B-ALL identified by S-LDSC. **Fig. S13.** Heatmap shows whether the identified risk loci have been reported to be associated with B-ALL and AD in the previous studies after searching the GWAS catalog.

**Additional file 2: Table S1.** Data sources. **Table S2.** Genetic correlation analysis conducted by LDSC and HDL. **Table S3.** Shared pleiotropic loci identified by PLACO. **Table S4.** Shared pleiotropic loci among different trait pairs. **Table S5.** MAGMA Gene-set analysis. **Table S6.** MAGMA tissue-specific analysis. **Table S7.** MAGMA gene analysis. **Table S8.** Information of pleiotropy genes identified by MAGMA. **Table S9.** Expression value of pleiotropy genes identified by MAGMA in different tissues from GTEx. **Table S10.** Tissue-specific enrichment of pleiotropy genes identified by MAGMA in different tissues from GTEx. **Table S11.** S-LDSC cell-type heritability enrichment analysis. **Table S12.** Multi-trait colocalization analysis highlighted key role of immune cells (PP>0.7). **Table S13.** Bi-direction MR analysis and sensitive analysis. **Table S14.** Bi-direction MR analysis and sensitive analysis. **Table S15.** Identified loci reported in previous GWAS analysis for ALL and AD.

## Data Availability

Data are available in public, open access repositories corresponding to the original studies (e.g., GWAS catalog). Main codes used in our research are available at https://github.com/biostatYu/MRcode/tree/main/AD_BALL.

## Abbreviations

AML: Acute myeloid leukemia
AOA: Adult-onset asthma
B-ALL: B-cell acute lymphoblastic leukemia
CD: Crohn’s disease
COA: Childhood-onset asthma
DEGs: Differentially expressed genes
DIVW: Debiased-inverse variance weighted
EBV: Epstein–Barr virus
eQTL: Expression quantitative trait loci
FUMA: Functional mapping and annotation
GD: Graves’ disease
GWAS: Genome-wide association study
HD: Hashimoto's disease
HDL: High-definition likelihood
HT: Hypothyroidism
HyPrColoc: Hypothesis prioritization for multi-trait colocalization
IBD: Inflammatory bowel disease
IUT: Intersection–union test
IVs: Instrumental variables
IVW: Inverse variance weighted
LD: Linkage disequilibrium
LDSC: Linkage disequilibrium score regression
MAGMA: Multi-marker analysis of genomic annotation
MR: Mendelian randomization
MR-PRESSO: Mendelian randomization pleiotropy residual sum and outlier
MR-RAPS: Mendelian randomization robust adjusted profile score
MS: Multiple sclerosis
MSigDB: Molecular signatures database
PBC: Primary biliary cirrhosis
PLACO: Pleiotropic analysis under composite null hypothesis
PSC: Primary sclerosing cholangitis
PVE: Proportion of variance explained
RA: Rheumatoid arthritis
SE: Standard errors
S-LDSC: Stratified-linkage disequilibrium score regression
SLE: Systemic lupus erythematosus
SMR: Summary-based Mendelian randomization
SS: Systemic sclerosis
T1D: Type 1 diabetes
UC: Ulcerative colitis
