# Supplementary material for: Shared genetic architecture between autoimmune disorders and B-cell acute lymphoblastic leukemia: insights from large-scale genome-wide cross-trait analysis
Source: BMC Med. 2024 Apr 15;22:161. doi: 10.1186/s12916-024-03385-0 (PMC11017616; doi:10.1186/s12916-024-03385-0)
Supplement: Supplementary file 1 — Additional file 1: Supplementary Methods and Fig. S1-S3. Supplementary Methods - A supplementary document on GWAS quality control, PLACO method, colocalization analysis, MAGMA analysis, HyPrColoc method, immune cell data description, and Mendelian randomization analysis. Fig. S1. Manhattan plot of the PLACO results. Fig. S2. -QQ plots for pleiotropic results performed by PLACO. Fig. S3. Regional plots of each colocalized locus (PP.H4 > 0.7) identified for corresponding trait pair (B-ALL&AOA) by using the PLACO. Fig. S4. Regional plots of each colocalized locus (PP.H4 > 0.7) identified for corresponding trait pair (B- B-ALL&HT) by using the PLACO. Fig. S5. Regional plots of each colocalized locus (PP.H4 > 0.7) identified for corresponding trait pair (B-ALL&PBC) by using the PLACO. Fig. S6. Regional plots of each colocalized locus (PP.H4 > 0.7) identified for corresponding trait pair (B-ALL&IBD) by using the PLACO. Fig. S7. Regional plots of each colocalized locus (PP.H4 > 0.7) identified for corresponding trait pair (B-ALL&MS) by using the PLACO. Fig. S8. Regional plot of each colocalized locus (PP.H4 > 0.7) identified for corresponding trait pair (B- B-ALL&RA) by using the PLACO. Fig. S9. Manhattan plot of MAGMA gene analysis. Fig. S10. Heatmap for expression values of pleiotropic genes in different tissues identified by MAGMA analysis. Fig. S11. Gene-set enrichment for identified pleiotropic genes. Red panels represent significant tissues after Bonferroni adjustment. Fig. S12. Heatmap of tissues and immune traits shared between autoimmune disorders and B-ALL identified by S-LDSC. Fig. S13. Heatmap shows whether the identified risk loci have been reported to be associated with B-ALL and AD in the previous studies after searching the GWAS catalog. [file 12916_2024_3385_MOESM1_ESM.docx]

## Additional file 1

## Supplementary Methods

##### GWAS data QC

We removed all non-biallelic SNPs, SNPs with chain ambiguous alleles (A/T, C/G alleles), SNPs with MAF <1%, and SNPs without rs IDs, duplicate SNPs, and SNPs whose alleles does not match Phase 3 of the 1000 Genomes Project. Additionally, SNPs located in major histocompatibility complex (MHC, chr 6: 25–35 Mb) region were excluded from main analysis and annotations due to its complex LD structure.

##### Pleiotropic analysis under composite null hypothesis analysis (PLACO)

This method could detect pleiotropic signals by using summary-level association statistics between complex traits. Considering the potential correlation among autoimmune diseases, we calculated correlation matrix of Z-scores. Then a level-α IUT method was used to test pleiotropy hypothesis: *H*_0_ is the null hypothesis, which could be expressed as $H_{0}:H_{00}\cup H_{01}\cup H_{02}$, and alternative hypothesis *H*_1_ could be further expressed as:

$$\begin{matrix} H_{1}:H_{00}^{a}\cap H_{01}^{a}\cap H_{02}^{a}\text{, } \\ \text{where }H_{00}:\beta_{AD}=\beta_{B-ALL}=0\text{,} \\ H_{01}:\beta_{AD}=0,\text{ }\beta_{B-ALL}\neq0\text{,} \\ H_{02}:\beta_{AD}\neq0,\text{ }\beta_{B-ALL}=0. \end{matrix}$$

The$H^{a}$represents the complement of *H*. $\beta_{Disease}$ represents effect size of autoimmune diseases. The maximum of *P* values for testing *H*_0_ vs *H*_1_ were viewed as the final *P* values.

##### Bayesian colocalization analysis using coloc

The coloc package can be used to perform genetic colocalization analysis of two potentially related phenotypes to test whether they share common genetic causal variants in given regions. The approach assumes that each genetic variant is equally likely to affect gene expression or a trait, and is only interested in whether shared causal variants are plausible. For different combinations of the two phenotypes, the study offers five hypotheses: ***H_0_***: No association with either trait; ***H_1_***: Association with autoimmune disorders, not with B-ALL; ***H_2_***: Association with B-ALL, not with autoimmune disorders; ***H_3_***: Association with autoimmune disorders and B-ALL, two independent SNPs; ***H_4_***: Association with autoimmune disorders and B-ALL, one shared SNP. Therefore, *H_4_* assumes that the effects of shared variants on two traits are independent, while high PP_4_ measures correlation, not causation.

##### MAGMA analysis

In MAGMA gene analysis, genetic marker data are aggregated to the gene level and converted from the association of test SNPs to the joint association of all markers in the test gene with the phenotype. The model for MAGMA gene analysis is based on the multiple linear principal component regression method, which uses the F test to calculate gene p-values. In order to ensure that the model is identifiable in the presence of highly collinear SNPs, the model projects the SNP matrix of the gene onto its principal component (PC). Then the PCs with very small eigenvalues were pruned, the remain PCs were viewed as predictor factors for phenotypes in the linear regression model. Likewise in MAGMA gene set analysis, individual genes are aggregated into genomes with certain biological, functional, or other characteristics. This aggregation has the advantage of greatly reducing the number of association tests that need to be performed and can detect effects consisting of multiple weak associations, greatly improving statistical power.

##### Multi-trait colocalization analysis using HyPrColoc

HyPrColoc is based on the similar statistical model as coloc, but unlike coloc, HyPrColoc uses summary statistics for a large number of traits to identify multi-traits colocalization association signals. This method accurately approximates the posterior probability of colocalization for a single causal variant by enumerating only a small number of putative causal associations (assuming that there is at most one causal variant per trait), avoiding repeated pairwise colocalization analyses, and identify co-localization signals between multiple traits efficiently and quickly. However, this method may increase the false negative rate and reduce the performance of identifying shared causal variants to some extent.

##### Detailed information of immune cells used in HyPrColoc analysis

The GWAS summary statistics for 731 immune traits could be publicly available in the GWAS Catalog (accession numbers from GCST0001391 to GCST0002121), of which 118 were absolute cell (AC) counts, 389 were median fluorescence intensities (MFIs) reflecting the levels of surface antigens, 32 were morphological parameters [MP, forward scatter (FSC) and side scatter (SSC), which are proportional to the cell volume, and intracellular complexity and the surface texture of cells, respectively], and 192 were relative cell (RC) counts. This GWAS analysis was conducted based on 3,757 European samples (57% women) to test around 22 million single nucleotide polymorphisms (SNPs) genotyped with high density arrays after adjusting for several covariates (i.e., sex, age, and age^2^). Finally, these SNPs were imputed with a sequence-based reference panel.

##### Detailed information of Mendelian Randomization methods

***Proportion of variance explained (PVE) and F statistic*** For a valid Mendelian stochastic analysis, each of the instrumental variables used for an exposure should simultaneously satisfy three modeling assumptions: (i) instruments should be related to exposure; (ii) instruments are independent of any confounding factors associated with exposure and outcome; (iii) instruments are unrelated to the outcome except by the pathway via exposure. In order to measure the strength of instruments, *F* statistic is constructed by formula:

$F=(\frac{n-1-k}{k})(\frac{\mathrm{PVE}}{1-\mathrm{PVE}})$,

where PVE represents the proportion of variance explained by genetic variants, *n* represents sample size, *k* represents number of SNPs.

***Cook’s distance*** We used Cook’s distance method to rule out possible outliers in MR analysis (i.e. large values indicate an influential effect of the *i*th observation). The distance *D_i_* is calculated by $D_{i}=H_{ii}r_{i}^{2}/(m(1-H_{ii}))$, where *H_ii_* is the *i*th element on the diagonal of the hat matrix, *m* is the number of parameters, *r_i_* is the standardized residuals of *i*th observation.

***Cochran's Q statistics*** We then employ the Cochran's *Q* statistics to examine the effect size heterogeneity across the instrumental variables and then the substantial heterogeneity can be calculated with

$I^{2}=100\times\frac{Q-df}{Q}$.

The low values of *I^2^* relate both to differences in predictive precision between MR-Egger and IVW estimates, and to weaker instrument bias.

***MR analysis*** In order to obtain accurate and reliable results, we excluded SNPs that reported to be related to outcomes in previous studies by screening the GWAS Catalog (<https://www.ebi.ac.uk/>). Missing instrumental variables in outcomes were imputed by the SNPs with the smallest *P* values in high LD regions. Fixed-effect inverse-variance weighted (IVW), random-effect IVW, weighted median-based method, debiased-IVW (DIVW) and MR-Egger regression, MR robust adjusted profile score (MR-RAPS), mode-based estimate were performed to examine the causal effects. Two IVW MR methods (fixed-effect and random-effect) were mainly conducted to estimate the causal effect of the exposure on the outcome based only on summary-level statistics. If heterogeneity exists in the effect sizes of the instrumental variables, random-effects IVW model was then used for estimation instead of fixed-effects. As a common sensitivity analysis, MR-Egger is usually used to test for directional pleiotropy and provides an estimate of the causal effect adjusted for its presence. Notably, the intercept of MR-Egger regression method was used as the main estimation to account for potential pleiotropy. Non-zero intercept term indicates an existence of potential pleiotropy. Besides, conventional IVW methods give consistent estimates only when all genetic variants in the analysis are valid, whereas the weighted median method is a novel estimator that is consistent even if up to 50% of the information comes from invalid instrumental variables. Another sensitive analysis MR pleiotropy residual sum and outlier (MR-PRESSO) method was utilized to validate possible horizontal pleiotropic outliers that affect the estimation substantially in the analysis.

# Supplementary Figures


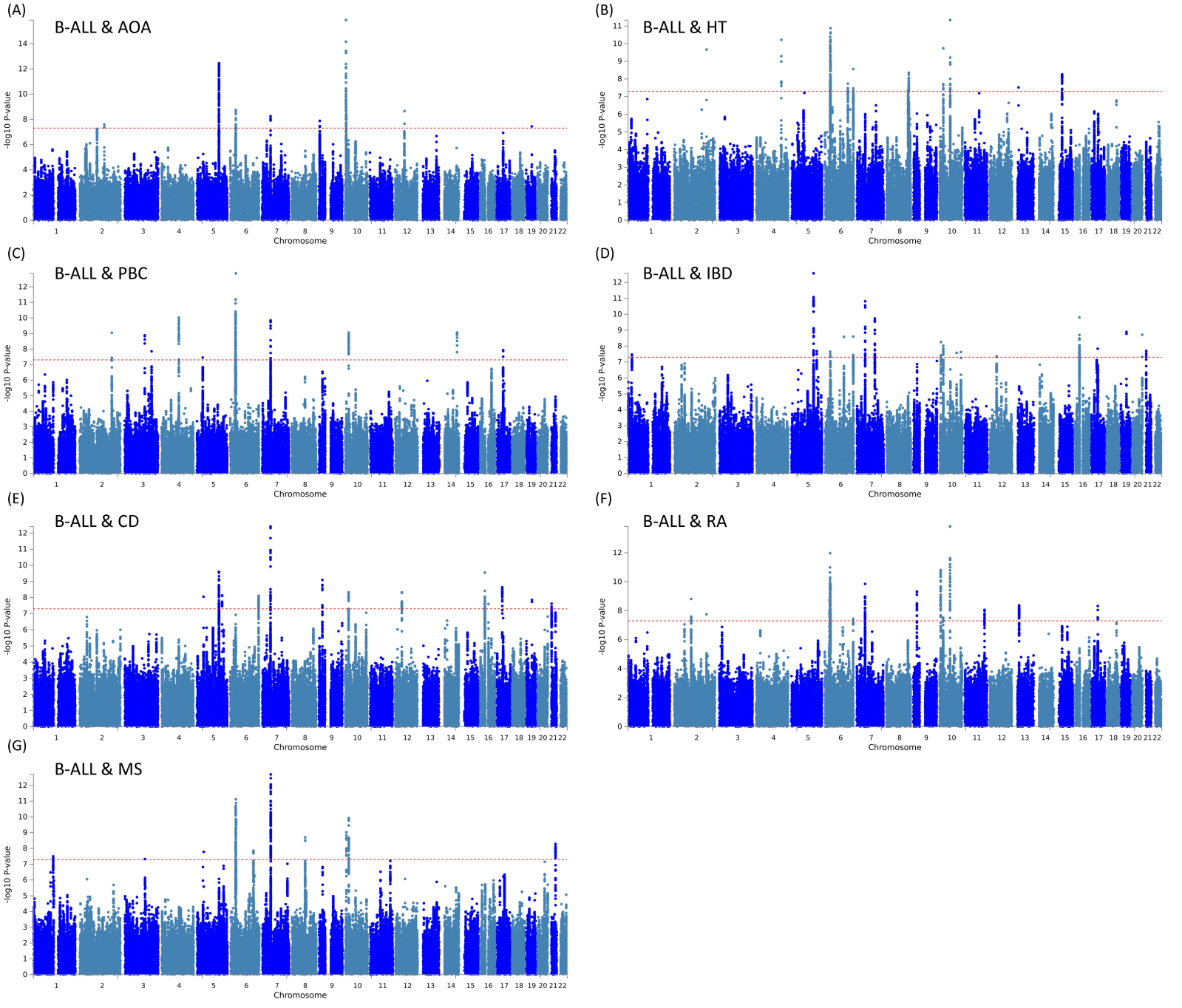


**Figure S1.** Manhattan plot of the PLACO results. Note: Red line represents the significance of 5×10^-8^. Abbreviations: B-ALL, B-cell acute lymphoblastic leukemia; AOA, adult-onset asthma; HT, hypothyroidism; PBC, primary biliary cirrhosis; IBD, inflammatory bowel disease; CD, crohn’s disease; RA, rheumatoid arthritis; MS, multiple sclerosis


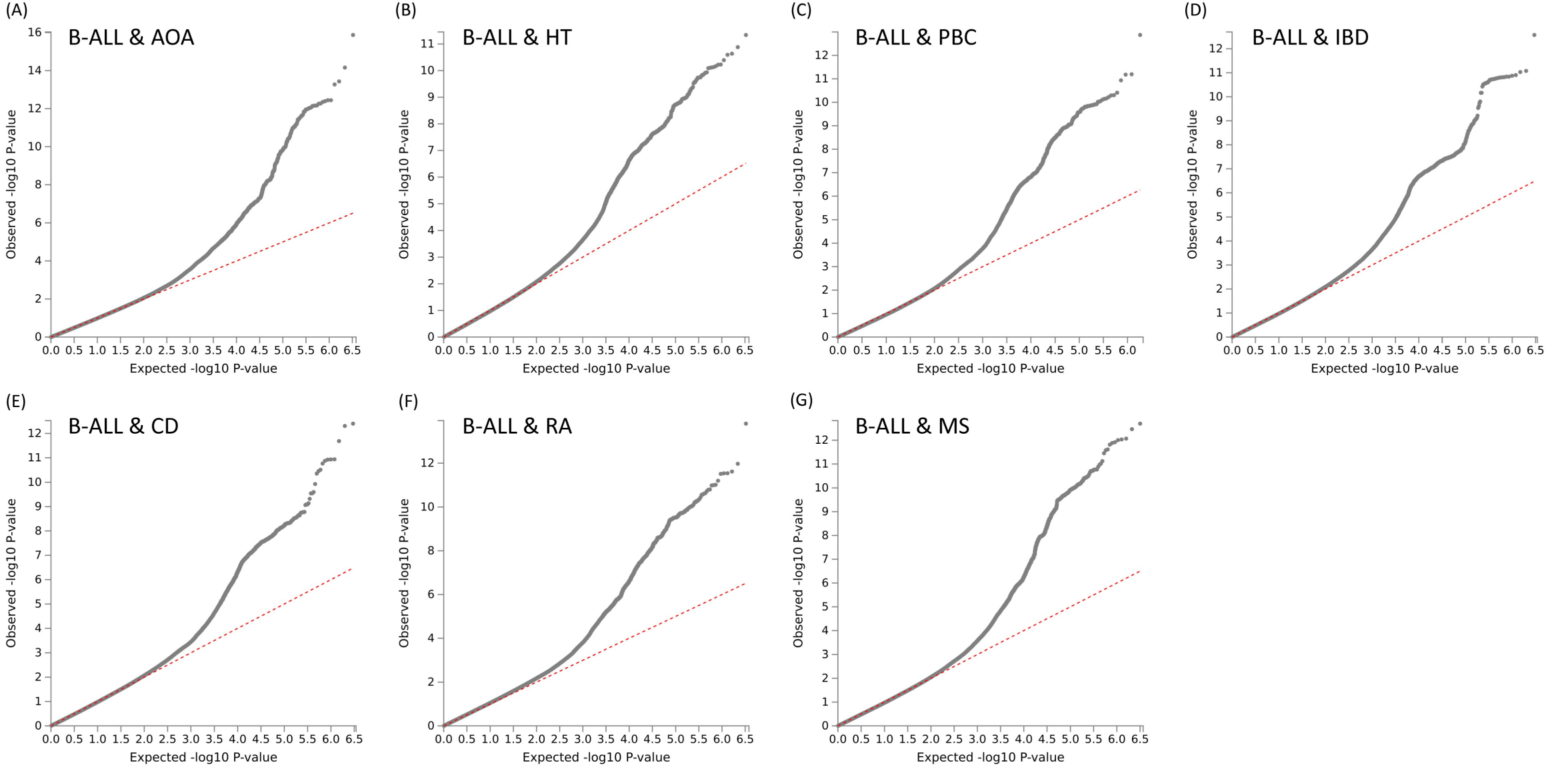


**Figure S2.** QQ plots for pleiotropic results performed by PLACO. Abbreviations: B-ALL, B-cell acute lymphoblastic leukemia; AOA, adult-onset asthma; HT, hypothyroidism; PBC, primary biliary cirrhosis; IBD, inflammatory bowel disease; CD, crohn’s disease; RA, rheumatoid arthritis; MS, multiple sclerosis.


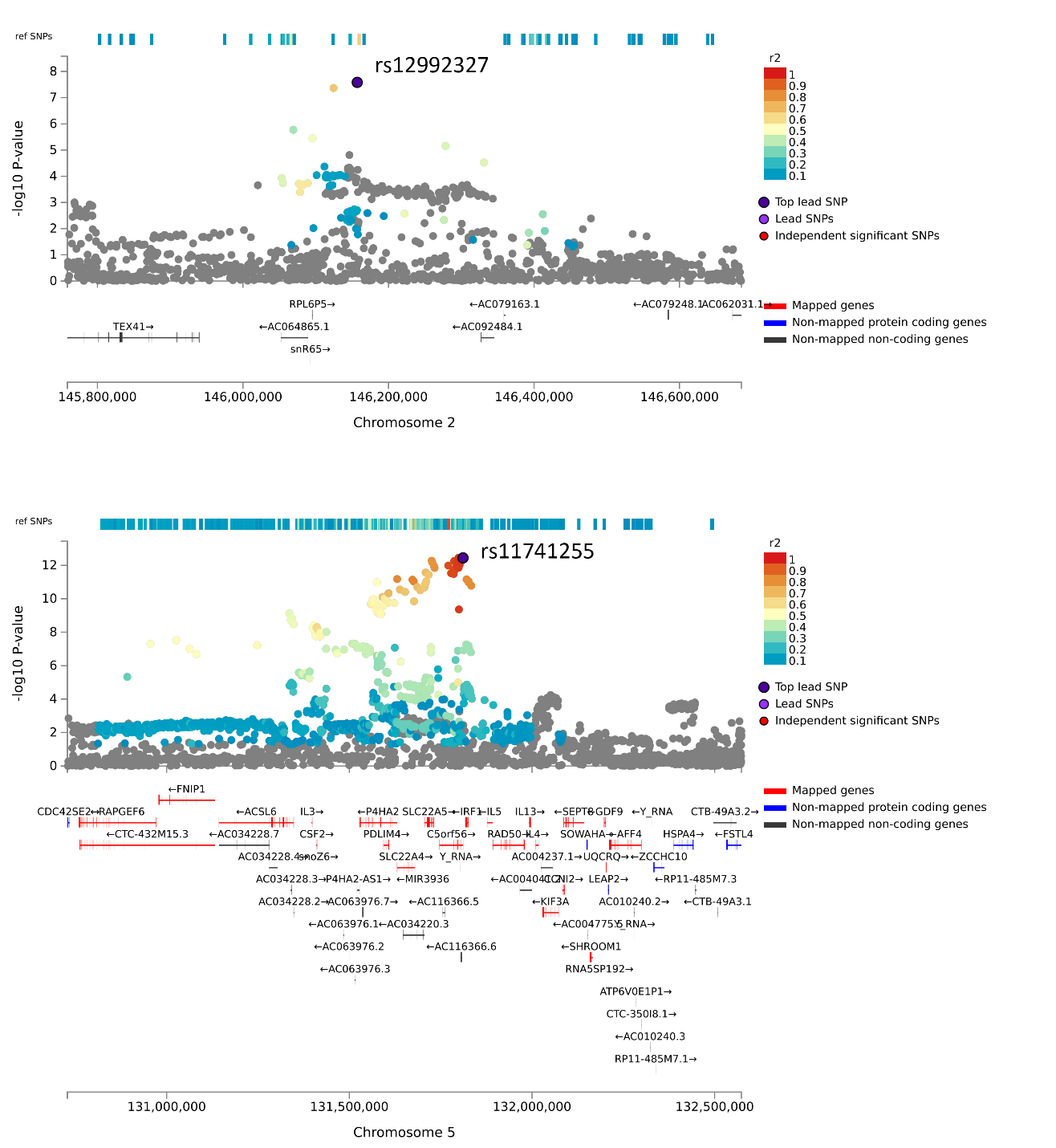


**Figure S3.** Regional plots of each colocalized locus (PP.H4 > 0.7) identified for corresponding trait pair (B-ALL&AOA) by using the PLACO. **Note:** PP.H4 was posterior probability of H4 calculated by coloc analysis; SNPs in LD that do not have any significant independent lead SNPs in the selected region are grayed out. For genes, mapped genes drawn by position mapping are in red; blue are unmapped protein-coding genes; dark gray are unmapped non-coding genes. Abbreviations: B-ALL, B-cell acute lymphoblastic leukemia; AOA, adult-onset asthma; SNP, Single-nucleotide polymorphism; LD, Linkage disequilibrium


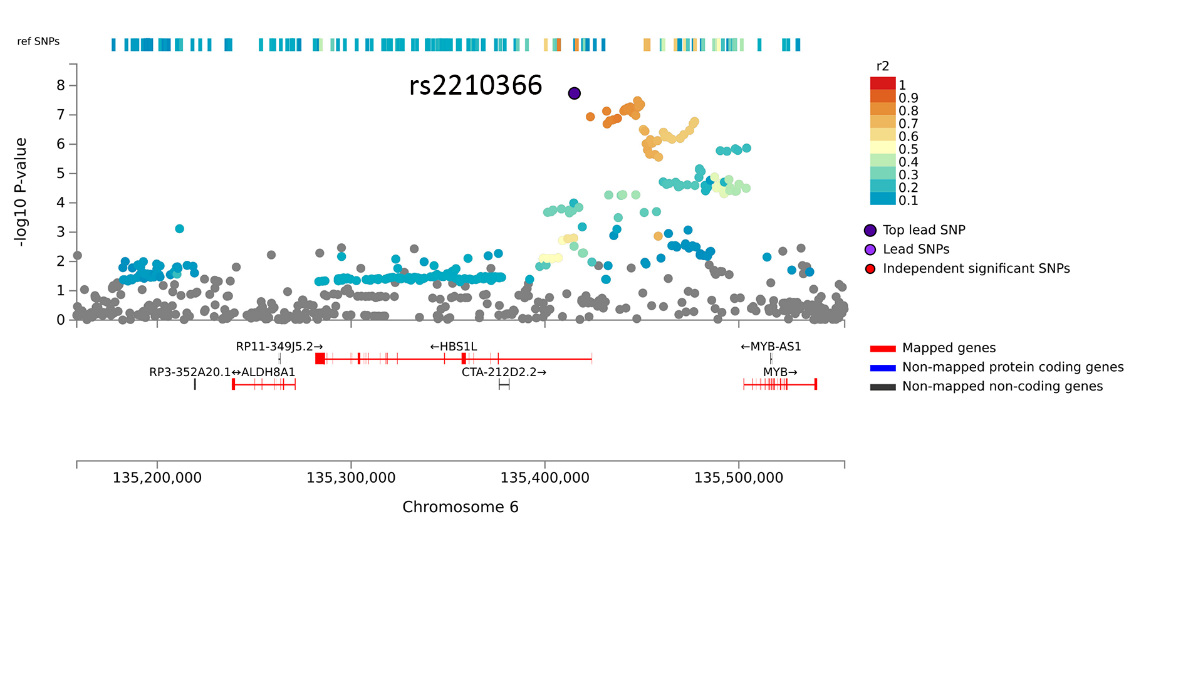


**Figure S4.** Regional plots of each colocalized locus (PP.H4 > 0.7) identified for corresponding trait pair (B- B-ALL&HT) by using the PLACO. **Note:** PP.H4 was posterior probability of H4 calculated by coloc analysis; SNPs in LD that do not have any significant independent lead SNPs in the selected region are grayed out. For genes, mapped genes drawn by position mapping are in red; blue are unmapped protein-coding genes; dark gray are unmapped non-coding genes. Abbreviations: B-ALL, B-cell acute lymphoblastic leukemia; HT, Hypothyroidism; SNP, Single-nucleotide polymorphism; LD, Linkage disequilibrium


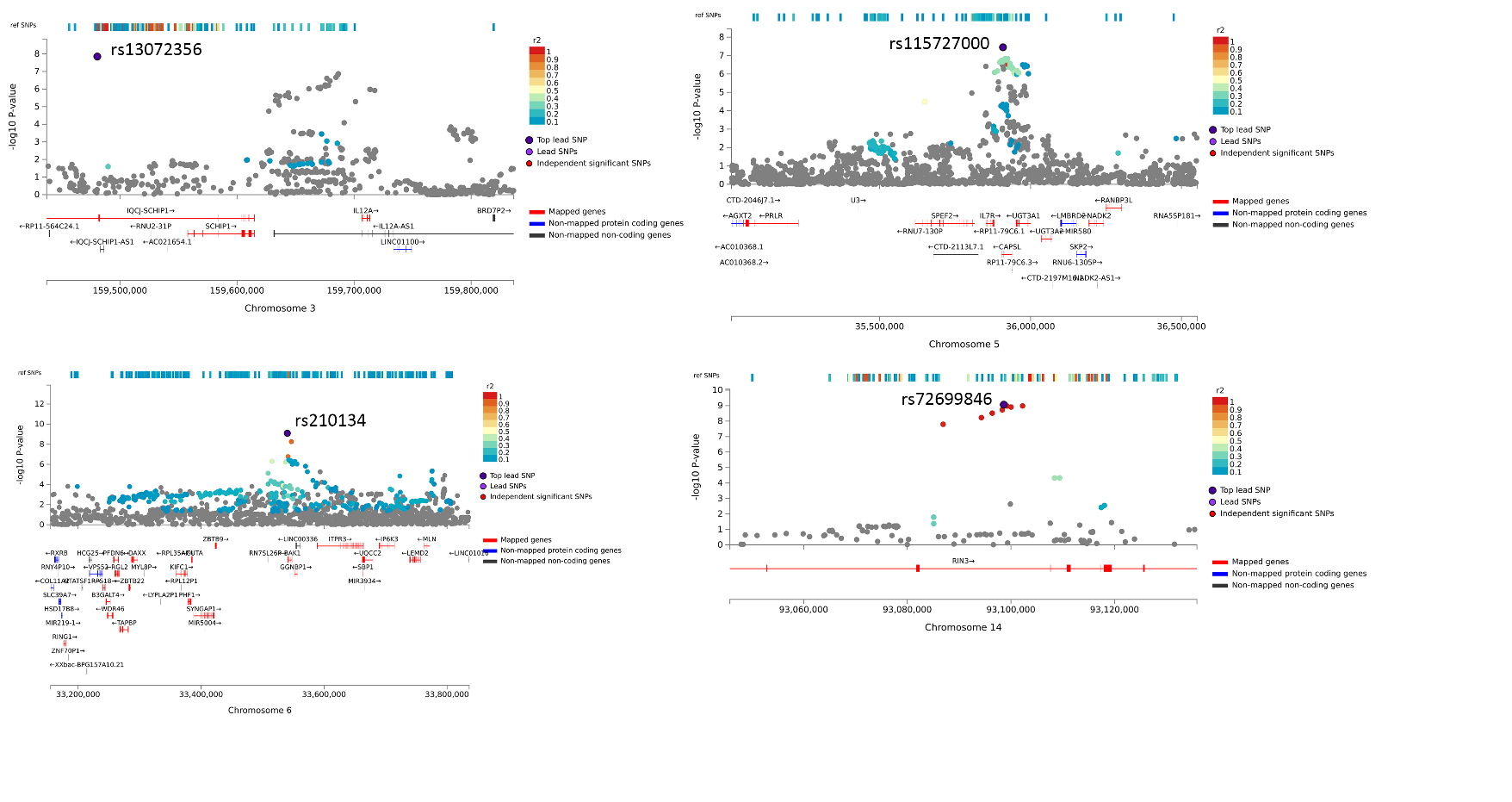


**Figure S5.** Regional plots of each colocalized locus (PP.H4 > 0.7) identified for corresponding trait pair (B-ALL&PBC) by using the PLACO. **Note:** PP.H4 was posterior probability of H4 calculated by coloc analysis; SNPs in LD that do not have any significant independent lead SNPs in the selected region are grayed out. For genes, mapped genes drawn by position mapping are in red; blue are unmapped protein-coding genes; dark gray are unmapped non-coding genes. Abbreviations: B-ALL, B-cell acute lymphoblastic leukemia; PBC, primary biliary cirrhosis; SNP, Single-nucleotide polymorphism; LD, Linkage disequilibrium


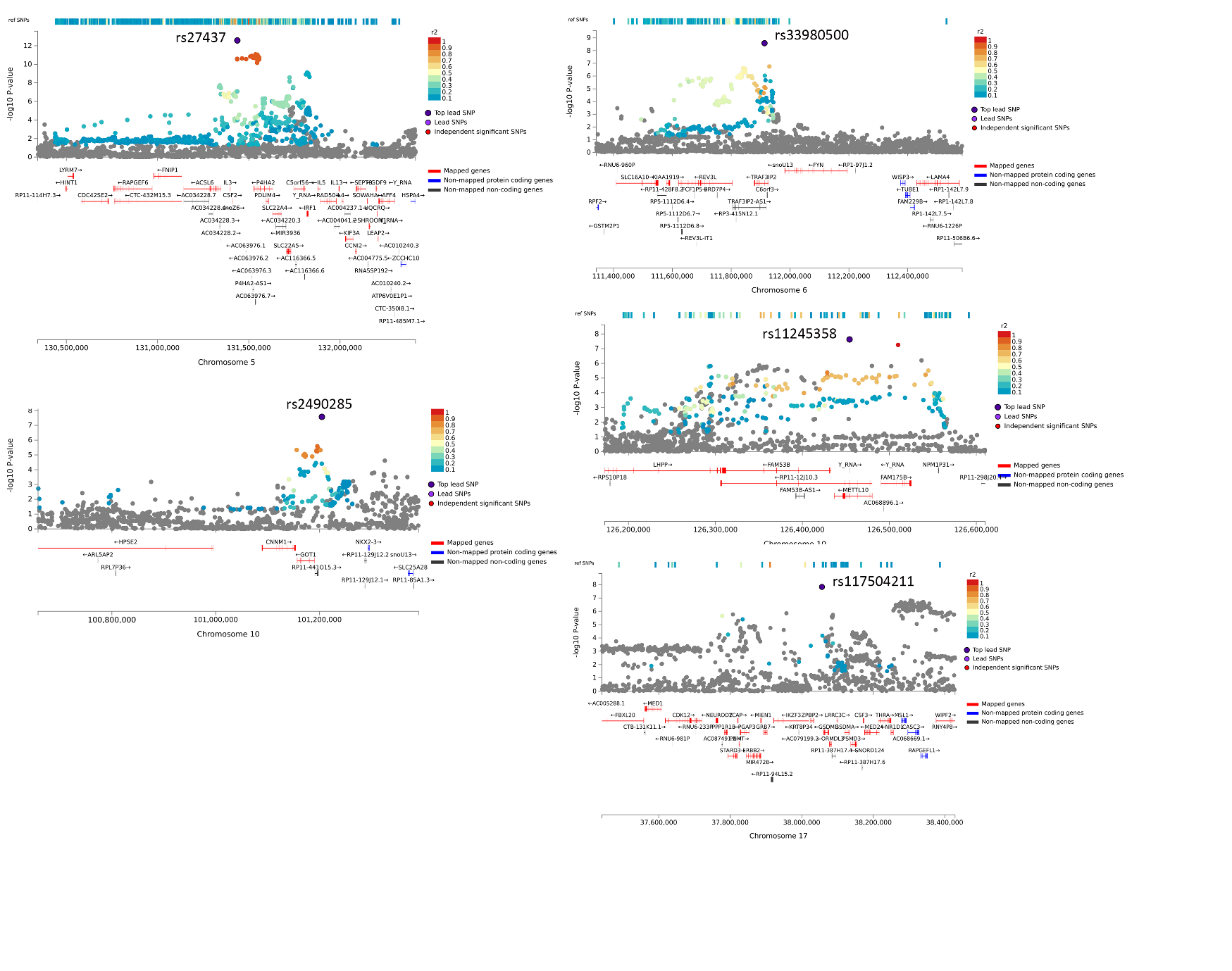


**Figure S6.** Regional plots of each colocalized locus (PP.H4 > 0.7) identified for corresponding trait pair (B-ALL&IBD) by using the PLACO. Note: PP.H4 was posterior probability of H4 calculated by coloc analysis; SNPs in LD that do not have any significant independent lead SNPs in the selected region are grayed out. For genes, mapped genes drawn by position mapping are in red; blue are unmapped protein-coding genes; dark gray are unmapped non-coding genes. Abbreviations: B-ALL, B-cell acute lymphoblastic leukemia; IBD, inflammatory bowel disease; SNP, Single-nucleotide polymorphism; LD, Linkage disequilibrium


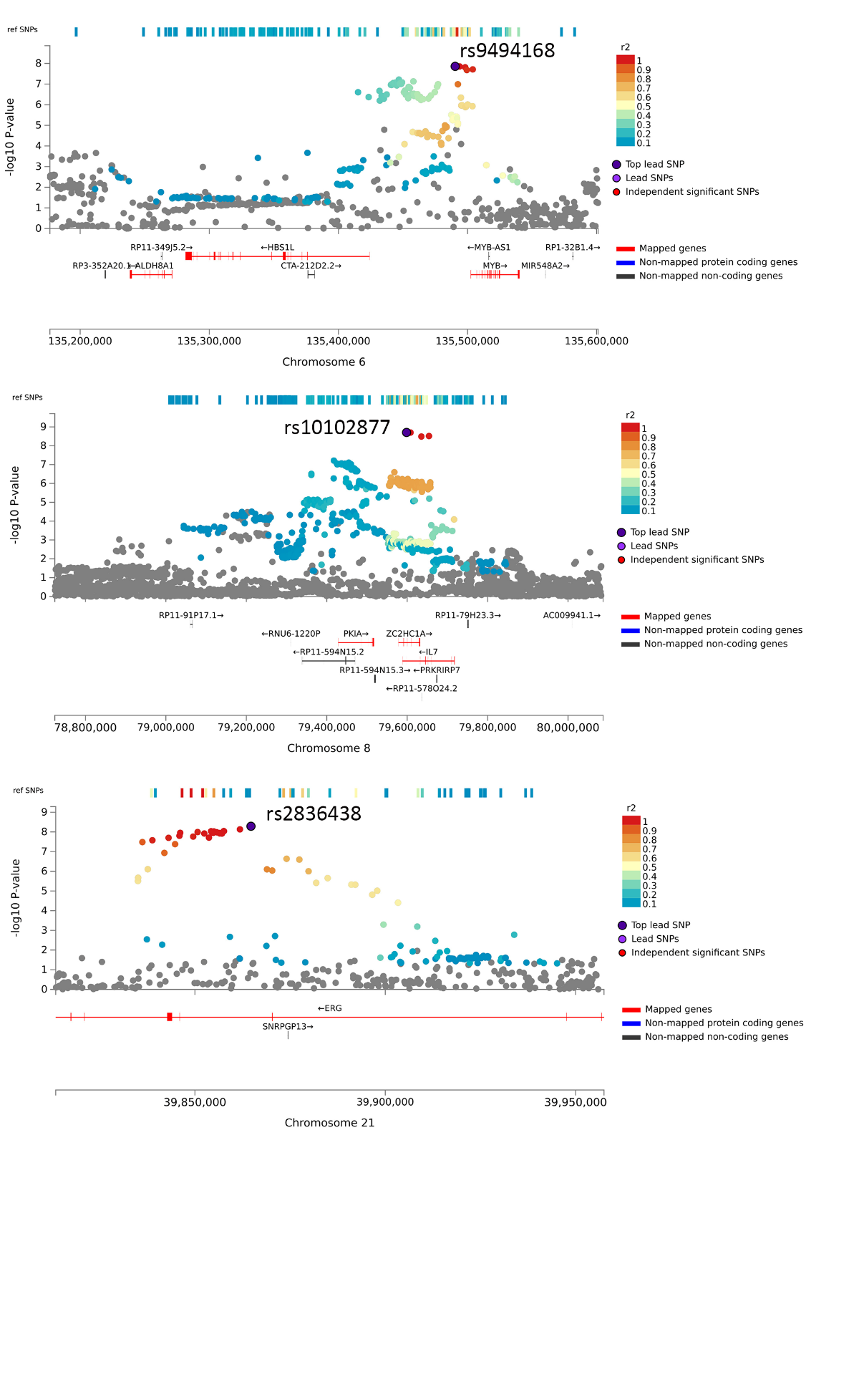


**Figure S7.** Regional plots of each colocalized locus (PP.H4 > 0.7) identified for corresponding trait pair (B-ALL&MS) by using the PLACO. **Note:** PP.H4 was posterior probability of H4 calculated by coloc analysis; SNPs in LD that do not have any significant independent lead SNPs in the selected region are grayed out. For genes, mapped genes drawn by position mapping are in red; blue are unmapped protein-coding genes; dark gray are unmapped non-coding genes. Abbreviations: B-ALL, B-cell acute lymphoblastic leukemia; MS, multiple sclerosis; SNP, Single-nucleotide polymorphism; LD, Linkage disequilibrium


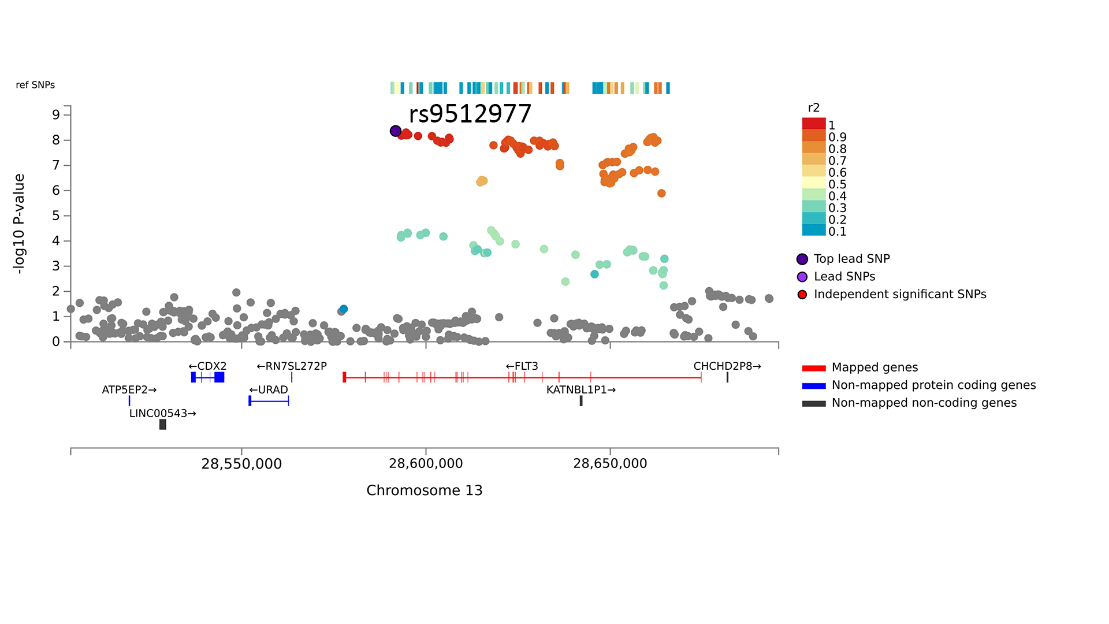


**Figure S8.** Regional plot of each colocalized locus (PP.H4 > 0.7) identified for corresponding trait pair (B- B-ALL&RA) by using the PLACO. **Note:** PP.H4 was posterior probability of H4 calculated by coloc analysis; SNPs in LD that do not have any significant independent lead SNPs in the selected region are grayed out. For genes, mapped genes drawn by position mapping are in red; blue are unmapped protein-coding genes; dark gray are unmapped non-coding genes. Abbreviations: B-ALL, B-cell acute lymphoblastic leukemia; RA, rheumatoid arthritis; SNP, Single-nucleotide polymorphism; LD, Linkage disequilibrium


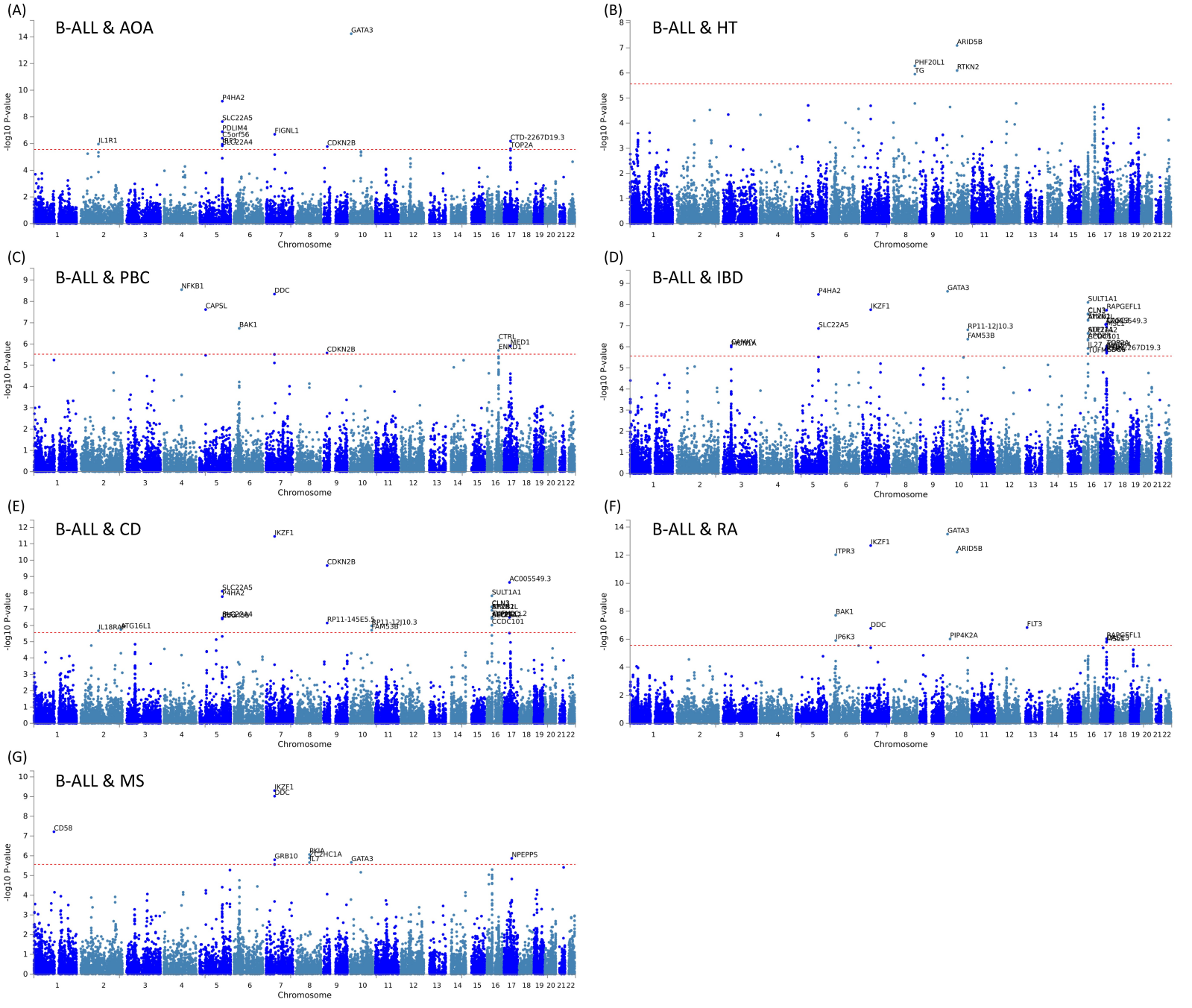


**Figure S9.** Manhattan plot of MAGMA gene analysis. Note: Red dot lines represent the Bonferroni significant threshold (*P* < 0.05/18,294) and the significant genes were labeled. Abbreviations: B-ALL, B-cell acute lymphoblastic leukemia; AOA, adult-onset asthma; HT, hypothyroidism; PBC, primary biliary cirrhosis; IBD, inflammatory bowel disease; CD, crohn’s disease; RA, rheumatoid arthritis; MS, multiple sclerosis


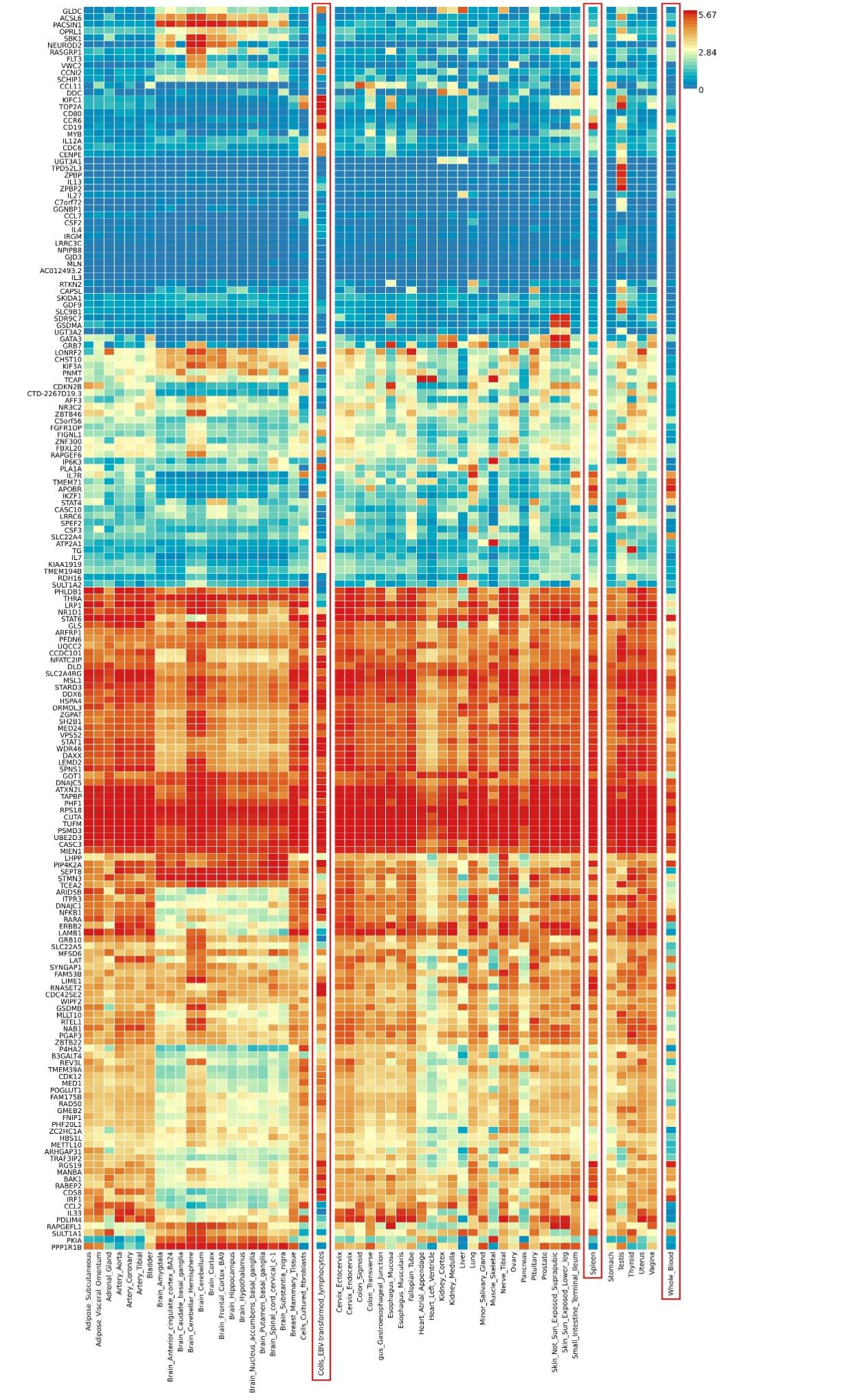


**Figure S10.** Heatmap for expression values of pleiotropic genes in different tissues identified by MAGMA analysis. Four tissues were highlighted in this figure.


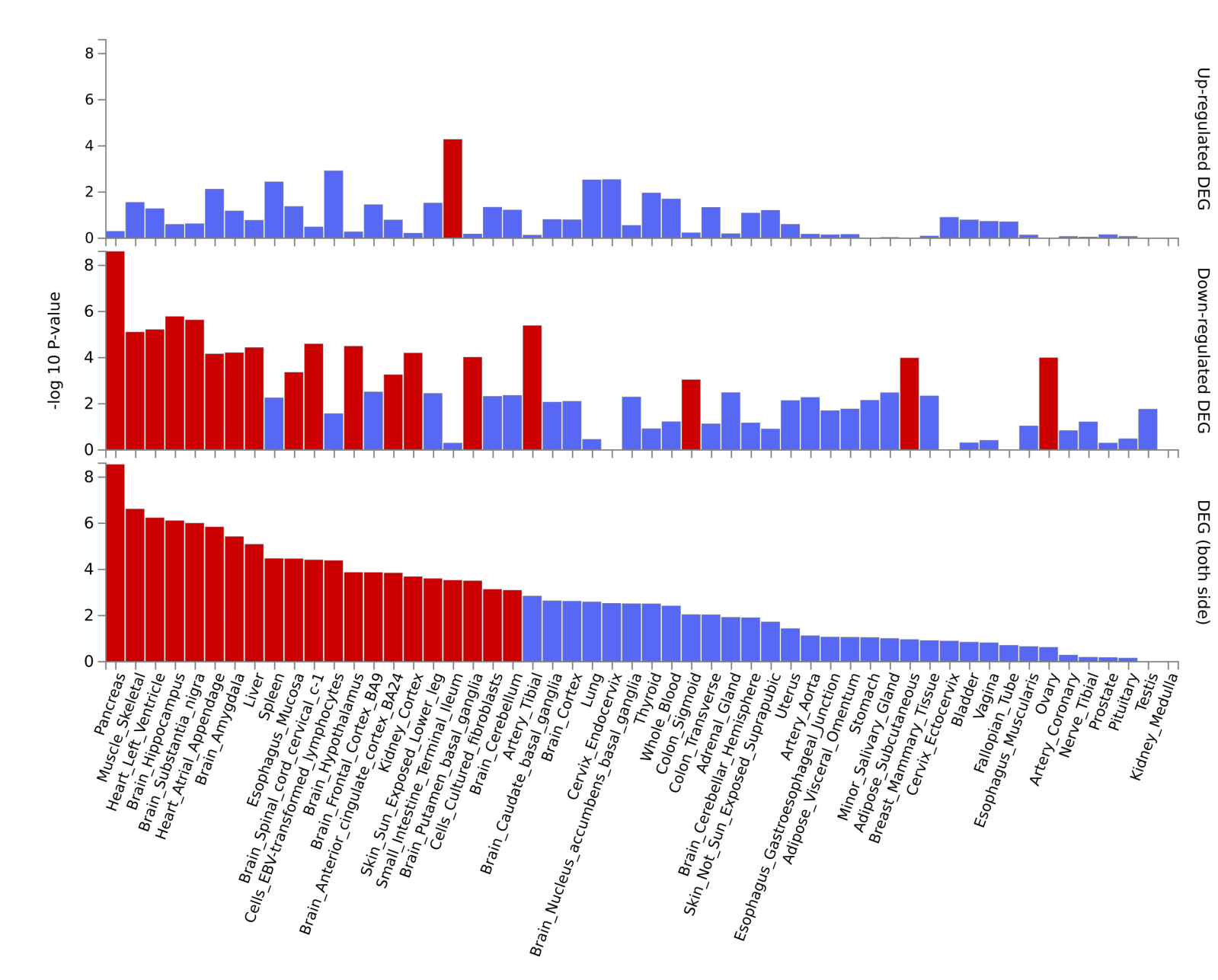


**Figure S11.** Gene-set enrichment for identified pleiotropic genes. Red panels represent significant tissues after Bonferroni adjustment. Abbreviations: DEG, differentially expressed genes.


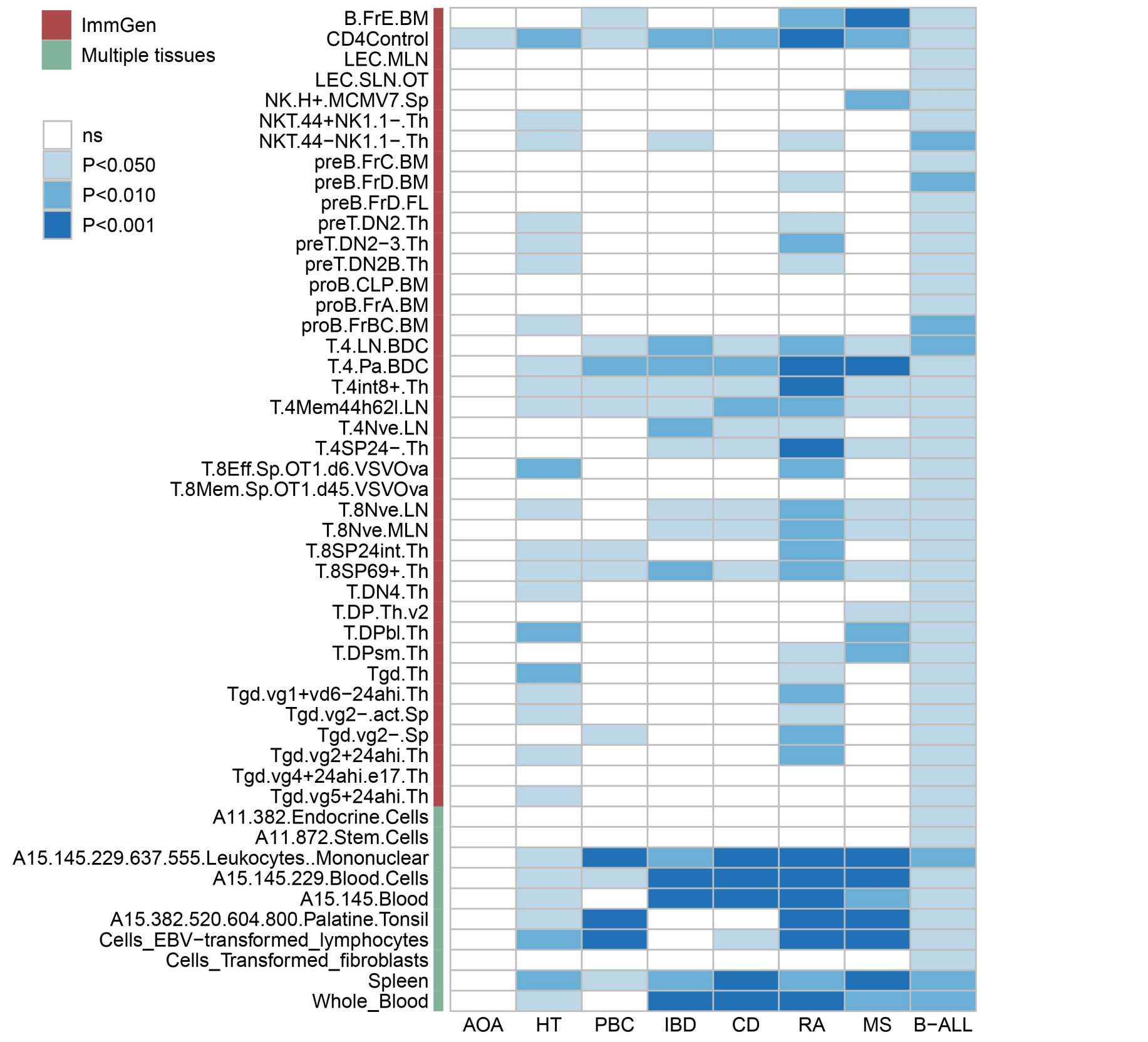


**Figure S12.** Heatmap of tissues and immune traits shared between autoimmune disorders and B-ALL identified by S-LDSC. Note: Red panel represents cell phenotypes from Immgen. Green panel represents tissues from GTEx and Franke lab. Abbreviations: B-ALL, B-cell acute lymphoblastic leukemia; AOA, adult-onset asthma; HT, hypothyroidism; PBC, primary biliary cirrhosis; IBD, inflammatory bowel disease; CD, crohn’s disease; RA, rheumatoid arthritis; MS, multiple sclerosis


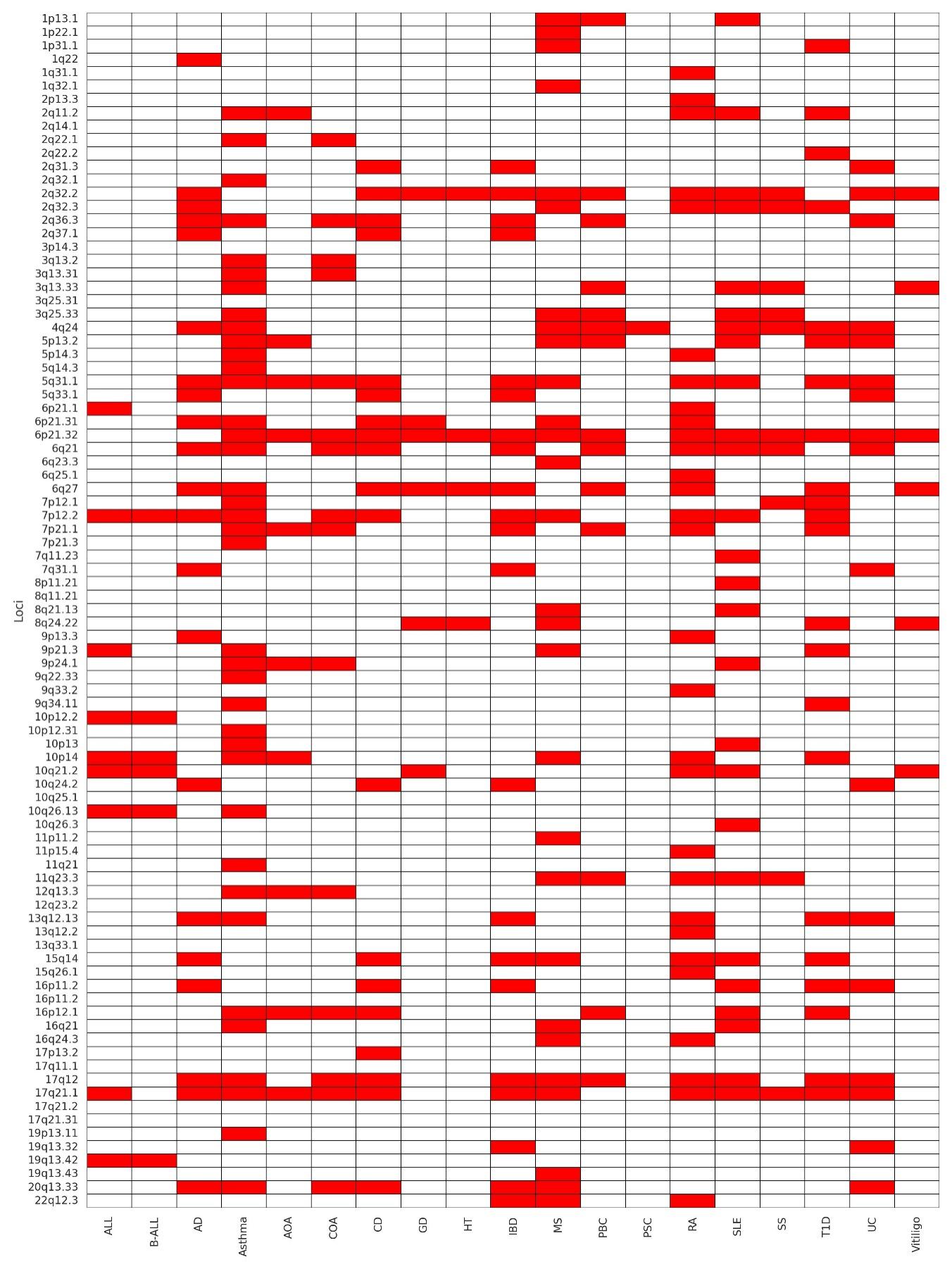


**Figure S13.** Heatmap shows whether the identified risk loci have been reported to be associated with B-ALL and AD in the previous studies after searching the GWAS catalog. Note: B-ALL, B-cell acute lymphoblastic leukemia; AOA, adult-onset asthma; COA, childhood onset asthma; GD, grave's disease; HD, hashimoto's disease; HT, hypothyroidism; PBC, primary biliary cirrhosis; PSC, primary sclerosing cholangitis; IBD, inflammatory bowel disease; CD, crohn’s disease; UC, ulcerative colitis; RA, rheumatoid arthritis; MS, multiple sclerosis; SS, systemic sclerosis; SLE, systemic lupus erythematosus; T1D, type 1 diabetes.
